# Correction: A behavioural model of minority language shift: Theory and empirical evidence

**DOI:** 10.1371/journal.pone.0254625

**Published:** 2021-07-09

**Authors:** The PLOS ONE Staff

A technical error that occurred during the production process resulted in this article’s peer review history publishing incorrectly; the publisher apologizes for the error. The full Responses to Reviewers from the peer review history can be found below.

## Supporting information

S1 File Response to reviewers, first revision. This file includes the authors’ response to reviewers for the first round of revisions.
(PDF)

S2 File Response to reviewers, second revision. This file includes the authors’ response to reviewers for the second round of revisions.
(PDF)
