# Supplementary material for: A behavioural model of minority language shift: Theory and empirical evidence
Source: PLoS One. 2021 Jun 4;16(6):e0252453. doi: 10.1371/journal.pone.0252453 (PMC8177434; doi:10.1371/journal.pone.0252453)
Supplement: S1 Appendix — (ZIP) [file pone.0252453.s001.zip › S1_Appendix.pdf]

S1 Appendix to the main text:

A Behavioural Model of Minority Language Shift: Theory and Empirical Evidence

José-Ramón Uriarte*and Stefan Sperlich†

May 20, 2021

1 Definition of Bilingual

The proportion of bilinguals in the three languages, denoted α, is obtained dividing the Number of Bilinguals by the Total Population. For Table 1, in the main text, it is the total population in the country. The definition of who is bilingual is set by the statistical authority in each country. They coincide in that it is the oral competence what determines whether an individual is qualified as bilingual or not. The proportion of bilinguals denoted α_i is obtained dividing the number of bilinguals of locality i by the total population of the locality (i.e., municipalities in the Basque Country, local electoral areas in Ireland, and local authorities in Wales).

2 Data Sources

- **Basque Country**: The data about knowledge of Basque can be found in the *Sociolinguistic Surveys (Inkesta Soziolinguistikoa).* These surveys are carried out by the local Government and agencies of each of the three territories where Basque is spoken: the Basque Autonomous Community (Spain), the Navarre Community (Spain) and Pays Basque (France). The population is divided in three groups according to the oral competence relative to Basque: Bilinguals (those who speak well or fairly well the language), Passive Bilinguals (those who do not speak well, but understand the language) and Monolinguals (of either Spanish or French). The *VI Sociolinguistic Survey 2016* gathered information

*University of the Basque Country, Departamento de Fundamentos del Análisis Económico I, Avenida Lehendakari Aguirre 83, E-48015 Bilbao, Basque Country-Spain.

†Geneva School of Economics and Management, Université de Genève, Bd du Pont d'Arve 40, CH-1211 Genève, Suisse.

from a sample of randomly chosen 8200 people, aged 16 and over, who answered a questionnaire over the phone. See the Basque Government's gathered data about Basque in: `www.euskara.euskadi.eus/r59-738/eu/contenidos/informacion/argitalpenak/eu_6092/ikuspegi_sozio_linguis.html`

The *Street Use of Basque Surveys* are carried out, every five years, by the NGO *Soziolinguistika Klusterra.* The street surveyors collect random observations, without any interference with the subjects observed, just by listening and recording the language of conversations taking place in the public spaces of the above mentioned three territories. The methodological details on how the data is collected are given in [1]. Note that the *V. Street Survey* in 2006 took place in 62 municipalities with 400 street surveyors recording 185,316 conversations. In the *VII. Street Survey* in 2016, 187,635 conversations in 144 municipalities were recorded. Using random samples of anonymously registered conversations in the streets at a given time and place, the *Street Use Measure* (*KE (i.e. Kale Erabilera)*) of minority language B is the number of individuals observed in conversations speaking language B out of the total number of individuals observed in conversations in that place. As a methodological rule, the observers only record conversations in a single language, cf. assumption **A.1** in the main text. Hence, if code-switching is observed, the conversation is not taken into account. The *street use* data can be found on `www.soziolinguistika.org`.

- **Ireland**: All data were obtained from the web-page of the Irish statistical institute, http://www.cso.ie/en/census/index.html. In all the censuses bilinguals are defined as "Irish speakers aged 3 years and over". The daily use, DU, is explicitly observed after 1996 separated into groups that daily use Irish "inside the educational system only" and those who also or exclusively use it outside the educational system. Certainly we consider the latter group. Since for 1996 this distinction is not at all clear we skipped this wave . Used sources are:

Census 1996. Volume 9-Irish Language.

Census 2002. Principal Demographic Results.

Census 2006. Volume 9-Irish language.

Census 2011. Profile 9. What we know.

Census 2011. This is Ireland.

- **Wales**: The most reliable source of data about Welsh speakers are the Censuses of Wales. It is not explicitly used the word bilingual; they consider "the number of people aged 3 and over able to speak Welsh". Under the heading of "able to speak Welsh" is included every person aged 3 and over who "Speaks, reads and writes Welsh" or "Speaks, reads, but does not write" or "Speaks but does not read or write". Data about the frequency of use of Welsh are in the WLUS (*Welsh Language Use Surveys).* The daily use, *DU,* is obtained dividing the number of "daily users" by the total population. Disaggregated data for daily use of Welsh by local authorities is obtained from Jones (2012). Used sources are:

The WLUS surveys:

- *The Welsh Language Use Surveys of 2004-06.* The Welsh Language Board. 2008. Cardiff.

- *Welsh Language Use in Wales 2013-15.* Welsh Government and Welsh Language Commissioner. 2015. Cardiff.

- Jones, HM (2012). *A Statistical Overview of the Welsh Language*, The Welsh Language Board. Cardiff.

The Censuses:

- *2001 Census: Report on the Welsh Language.* Office for National Statistics, London: TSO. 2004.

- *2011 Census. First results on Welsh*, Statistical Bulletin 118/2012. Statistics for Wales.

Discrepancies between the Welsh language sources: For Welsh there are two authoritative sources of information for the period under consideration. The 2001 and 20011 Censuses provide information about the proportion of people aged three and over living in Wales who speak Welsh; that is, information about the country 's α and each local authority's α_i. The WLUS surveys provide information from the Welsh speaking people to ascertain, among other things, how often they speak Welsh; that is, information about the country's daily use DU and the local authorities' DU_i. The surveys reported that the DU kept constant at 13 per cent in the 2004-06 and 2013-15 periods (as shown in Table 1 of the main text). However, the Censuses and the WLUS surveys disagree about α. While the Census reported a decrease from $\alpha = 20.8$ in 2001 to $\alpha = 19.0$ in 2011, the surveys report an increase from $\alpha = 20.5$ to 24.0. In Table 1 we report the figures of α according to the Census, and those of DU according to the surveys: $(20.5 \times 63)/100 = 13.0$ for the mid-term year 2005 and $(24.0 \times 53)/100 = 13.0$ for 2014, where 63 and 53 are the percentages of Welsh speakers who speak Welsh daily in Wales according to the WLUS (see Table 3 in WLUS 2013-15). The sections of Fig 2 in the main text corresponding to Welsh and Fig S3 in the present SI are based on [2], Jones (2012) who reports the WLUS data.

DATA SET: We may send on request two *Excel files* with data about Irish and Basque. In the latter case, the headings were written in Basque with English acronyms by the surveyors who collected the data. The headings in the columns are: Year (1991, 1996, 2001, 2006, 20011 and 2016 when the *Sociolinguistic Survey* was made), Municipality, Bilinguals (Euskaldunak), Passive Bilinguals (Ia Euskaldunak), Monolinguals (Erdaldunak); then come the percentages of Bilinguals, Passive Bilinguals, and Monolinguals. Year (1993, 1997 2001, 2006, 2011 when the *Street Surveys* were made), Street Use of Basque (Euskeraren Kale Erabilera). In the 2016 Excel file the headings are: Municipality (Udalerria), Year of the Survey (Neurketa), Bilingual (Gaitasuna), Sample Size (Lagina), Street Use of Basque (Euskeraren Kale Erabilera).

3 Linguistic Information in Modern Bilingual Societies

Under imperfect information bilinguals do not recognize each other, creating uncertainty about which language one should use in the interaction. Evidence that imperfect information about individual linguistic type is a real problem in the bilingual societies under

study are the measures taken to avoid it. For instance, Welsh speakers may wear the Cymraeg badges of the *Welsh Language Commission* [3], so that bilinguals could recognize each other. Wearing badges could have been politically controversial in the Basque Country when the terrorist group, ETA, was active. But this is not the case nowadays. The cultural activist group *Euskaraldia* [4] has become successful in distributing two different badges. One shows that you want to be addressed in Basque (you understand the language, but you might have difficulties in speaking it). The other badge shows that you want to speak in Basque, so you will utter your initial words in Basque.

The main sources of imperfect information about linguistic types are the following:

1. **The process of modernization**: A feature of modern societies is the mobility, social and geographical, of the work force, and the growing of metropolitan cities and city regions, where new technologies and production systems are located. More than half of Basque speakers live in the six most populated towns. Similarly, 75.7% of Welsh speakers live in towns of more than 100.000 inhabitants; 64.8% of Irish speakers live in urban areas, and are considered to be the future support of the language. In these benchmark bilingual societies, the spread of bilinguals across all the layers of society would mean that frequently bilinguals participate in interactions without recognizing each other, because the linguistic type of individuals has become private information. That is, in modern urban contexts, interactions tend to be anonymous.

2. **Politeness behaviour**: The implementation of strategy R demands always full awareness in leading a conversation (which does not necessarily mean speaking first) to discover the interlocutor's linguistic type. When a monolingual is addressed in B, she is forced to reveal her type, and confess her lack of knowledge of the official language B. However, the need of communication will frustrate the bilingual's desire of using B, and is also forced to speak in A. This might create a tension between the interactants (not free of political undertones, in certain cases) that could interfere the final goal of the interaction. The linguistic distance between A and B (assumption **A.1**) is a source of that conflict. Then, given the bilinguals' linguistic flexibility, a growing proportion of them tend to choose the less demanding strategy H (addressing first in language A, or answering in the language used initially by the interlocutor) to avoid potential tensions. It is mistakenly argued that it is not *polite* to use, or signal the wish to use, B with unknown interlocutors. The politeness induced behaviour leads to the strategy of hiding the bilingual type and, hence, of reinforcing the imperfect information. Hence, by choosing H bilinguals become disguised monolinguals. The subtleties of this potential conflict and tension could be analysed by means of *linguistic politeness theory.*

3. **Additional sources:**

(a) the tight language contact and interaction with monolingual speakers erase some revealing signals of B speakers, such as the accent. Both bilinguals and monolinguals tend to have a similar accent, which is shaped by the dominant language (for example, in the Spanish side of the Basque Country, bilingual and monolingual people have similar Spanish accent, while in the French side they have a French accent); (b) Second and third generations of immigrants learn language B in the public educational system. Thus, differences in ethnic

features, if any, hardly reveal the linguistic type.

Thus, imperfect information on linguistic type is the result of a combination of several factors. To conclude, we think it is realistic to assume that in advanced bilingual societies individual linguistic type is *private information.* However, since sociolinguistic surveys are periodically published and talked about in the media, bilinguals know the proportions α_i and $1 - \alpha_i$.

4 Linguistic Reference Points, Aspirations, Linguistic Preferences, Payoffs and Strategies: a Behavioural Approach

We develop here a theory based on fundamental concepts of behavioural economics. We would have preferred to have all the data to test each of our arguments, but this is a problem shared by, probably, all papers testing theory with data. Hence, one can only test the resulting model. Our theory assumes that each bilingual individual is adapted to a sociolinguistic context. The intuition is based on Kahneman and Tversky's psychological *reference point* [5], which is a cornerstone in behavioural economics:

"Our perceptual apparatus is attuned to the evaluation of changes or differences rather than to the evaluation of absolute magnitudes. When we respond to attributes such as brightness, loudness, or temperature, the past and present context of experience defines an adaptation level, or reference point, and stimuli are perceived in relation to reference point [...]. Thus, an object at a given temperature may be experienced as hot or cold to the touch depending on the temperature to which one has adapted. The same principle applies to non-sensory attributes such as health, prestige, and wealth."

We apply this idea to the perception of bilinguals about the minority language B. In a language contact situation between a minority language and a majority one, the frequency with which a bilingual experiences the event of meeting another bilingual and speaking in B determines a *linguistic adaptation level* or *linguistic reference point.* This adaptation conditions the bilingual's expectations of the same event in the future. It will condition her language choice behaviour, her experienced utility of speaking B, and the aspirations concerning language B. Each locality where B is spoken will shape a specific *linguistic reference point* for the bilinguals of the locality.

A locality (i.e., municipality or local authority) is a sociolinguistic context i characterized by a specific proportion of bilinguals, α_i, and a certain use and presence of language B in the workplace, streets, etc. (i.e., each locality has its own linguistic landscape [6]). For instance, the sociolinguistic context of the heavily industrialized metropolitan area of Bilbao (23% of Basque speakers and a KE of 2.5% in the 2016's survey) is quite different from that of, say, San Sebastian-Donostia (with 40.6% and 15.2%, respectively in the 2016's survey). The same applies to the context of Dublin city and its suburbs (35.2% of Irish speakers, and a DU of 1.3%, in the 2011 Census) relative to the towns and villages of the Gaeltacht areas, such as Galway and its suburbs (44.2%, and 3.3%, respectively, in the 2011

Census). Similarly, Cardiff (11.1% and 8.13%, respectively, in the 2011 Census, and Survey of 2013-15) relative to Gwynedd (65.4% and 62.17%, respectively; same sources).

For each of the considered languages, there is a rich set of values of α_i, representing the variety of sociolinguistic contexts for B, $i \in \{\textit{all localities where B is spoken}\}$, each defining a linguistic reference point for the bilingual inhabitant of locality i.

To simplify notation, there is no confusion in not using a subindex to refer to each of the three B languages, since the base model is the same for all.

4.1 Bilinguals' Aspirations

Language A has no impediments to its use because we have assumed it is an official language and every individual in the society, either monolingual or bilingual, speaks A with similar competence and skill. That is, A is a fully normalized language. Bilinguals know that that state of A is unreachable for language B. However they aspire, more realistically, to B becoming a non-endangered language and to minimize the impediments to its use. Both goals imply a certain percentage of bilinguals in nearly all the localities of the country, higher than the actual percentages.

Let us consider any of the three mentioned B languages (Basque, Irish and Welsh), and let i be a locality with a proportion α_i of B speakers, $i \in \{\textit{all localities where B is spoken}\}$. We may think that for the bilinguals of i there is a hypothetical proportion of B speakers with which language B would be out of danger, and almost normalized, both at the local and at the country levels. This hypothetical proportion of bilinguals will be the *aspiration* level for the bilinguals of i, which will be somehow conditioned by the *linguistic reference point* for the bilinguals of i (i.e. the actual proportion α_i and use of B at i). However, there would be a kind of unanimity on aspirations, since no noticeable disagreements would exist between the aspirations from different localities. More specifically, let $S(\alpha_i) = \alpha_i^*$ be the *aspiration function* that assigns to each α_i, the aspiration level α_i^*, where $0 < \alpha_i < \alpha_i^* < 1$. We assume that S is an increasing and concave function in α_i, with a curvature that arranges all local aspirations into a similarity interval (that is, the curvature of S is almost flat). Let $\underline{\alpha}_q^*$ denote the locality q with the lowest aspiration level, and $\overline{\alpha}_z^* < 1$ the locality z with the highest aspiration; $q, z \in \{\textit{all localities where B is spoken}\}$. We assume that $\underline{\alpha}_q^*$ and $\overline{\alpha}_z^*$ are approximately the same and hardly distinguishable; that is, they are similar, $\underline{\alpha}_q^* \simeq \overline{\alpha}_{iz}^*$ (where $\simeq$ denotes the binary relation "similar to"). The unanimity reflected by the similarity between the local aspirations would allow the language policy authorities to reach a consensus in setting a value for the *country's aspiration level,* denoted α^*, with $\alpha^* < 1$. We assume they choose α^* so that $\alpha^* > \overline{\alpha}_{iz}^*$, and similar to both $\underline{\alpha}_q^*$ and $\overline{\alpha}_{iz}^*$: $\alpha^* \simeq \underline{\alpha}_q^*$ and $\alpha^* \simeq \overline{\alpha}_{iz}^*$. Then $\left[\underline{\alpha}_q^*, \alpha^*\right]$ is a similarity interval, with α^* being the greatest of the similar to $\underline{\alpha}_q^*$. By the *Betweenness* property, all the local aspirations in the interval are similar to each other (see [7] for the properties of similarity relations). That is, for all α_i, $S(\alpha_i) = \alpha_i^* \simeq \alpha^*$, with $0 < \alpha_i < \alpha_i^* \simeq \alpha^* < 1$.

Notice that some locality i's aspiration α_i^* might coincide with the actual proportion α_j of some locality j, $i \neq j$.If α^* were reached, the authorities, and the whole society, will agree

that B would be already a non-endangered, and "almost" normalized language.

In a choice situation, the *aspiration* represents the most desired alternative, *available or not*. It may happen that for a small community of bilinguals of some locality i, α^* is not feasible or is out of reach, due to its cultural and sociological constraints. However, it is well known, experimentally and theoretically, that unavailable choices have an important influence on the decision behaviour of agents, known as *aspiration effects* [8, 9].

4.2 Bilinguals' Utilities and Linguistic Preferences

1. **Payoff n**: we can think of $n > 0$ as a given constant, a kind of "natural" payoff obtained from using voluntarily the normalized language A. Hence monolinguals will get payoff n. Similarly, since language choices are made under imperfect information, a bilingual may choose voluntarily language A, and will get, as well, payoff n.

2. **The m and c payoffs as functions of α_i**: $m(\alpha_i)$ and $c(\alpha_i)$.

- **Localities with low α_i**: Let $\alpha_i < 20\%$; then the probability of matching between bilinguals is small, and so the probability of speaking in B could even be smaller. The bilinguals of those contexts will be adapted to those frequencies. But, at the same time, since the distance between the actual α_i and the aspiration level α^* is high, the *aspiration effect, when it happens, will be high.*

The weak social use of B, and the overwhelming presence of A, will weaken the language skills and competence in B of a relevant proportion of bilinguals. Due to the contact situation, language B becomes the recipient of the language structures of A, and when bilinguals interact with each other, they either speak A because of imperfect information, or they often code-switch between A and B. As a consequence, a proportion of the flow of potential bilinguals, produced by the educational system, will be lost. However, some bilinguals will feel high levels of frustration, c, witnessing the fading away of B. Then they will take actions to avoid the low bilingual matching probabilities by building ways to meeting other bilinguals and satisfy their wish of speaking in B. They will make the effort of creating clubs of B speakers, spend personal time in organising activities (music gigs, drama productions, talks, sports, language teaching for adults, and other events), just for the pleasure of speaking B, and feeling that they are part of a cultural identity. These types of clubs abound in the countries under study: in the Basque Country they take the name of *Mintzalagunak* "Speak with Friends"; in Ireland are called *Na Gaeil Óga GAA Club*; and in Wales *Mentrau Iaith Cymru.* Clearly, the experienced utility felt by these bilinguals when they speak B, denoted m, must be high. This is the result of *the aspiration effect.*

- **Localities with high α_i**: Let $\alpha_i \geq 60\%$; then the distance between the actual α_i and the aspiration α^* would be small, and therefore *the aspiration effect will be weak.* In contexts where the bilinguals are a majority of the population, the event of two bilinguals matching and speaking in B is fairly frequent. Thus, bilinguals will be adapted to those frequencies. If B is the dominant language of communication, then the *perceptible utility gain* that bilinguals would experience from speaking B, m, cannot be far from the payoff, n, obtained from using voluntarily the normalized language A. Further, the bilingual would

not suffer a *perceptible utility loss* due to being forced to use A when meeting, occasionally, a monolingual. That is, we could assume that c would be near zero in these contexts.

All this suggests that m and c are functions of α_i -denoted $m(\alpha_i)$ and $c(\alpha_i)$, respectively- whose domain is the set of all proportions α_i, one for each locality. These two functions satisfy the following properties or assumptions:

Assumptions: Let α_i denote the proportion of B speakers in locality i reported in the linguistic survey of a given year; $i \in \{\textit{all localities where } B \textit{ is spoken}\}$.

A.3.1. *The experienced utility obtained from using* B *is a real valued function,* $m(\alpha_i) > 0$, *that strictly decreases with* $\alpha_i \in (0, \alpha^*)$. *As* α_i *tends to the country's aspiration* α^*, $m(\alpha_i)$ *tends to the payoff* $n > 0$.

A.3.2. *The frustration cost of being forced to use* A *when matched with a monolingual is a real valued function,* $c(\alpha_i) > 0$, *that strictly decreases with* $\alpha_i \in (0, \alpha^*)$. *As* α_i *tends to the country's aspiration* α^*, $c(\alpha_i)$ *tends to zero.*

A.3.3. *Given* $\alpha_i < \alpha^*$, *the bilinguals of locality* i *strictly prefer to use (or speak)* B *rather than* A: $m(\alpha_i) > n$, *where* $n > c(\alpha_i) > 0$, *and* $c(\alpha_i) < (m(\alpha_i) - n)\frac{\alpha_i}{(1-\alpha_i)} =: b(\alpha_i)$ *(weighted benefit).*

Corollaries:

1. Decreasing preference intensity for using language B: by **A.3.3**, bilinguals prefer to use language B, but, by **A.3.1**, the preference intensity, shown by $m(\alpha_i) - n$, decreases with the increase of α_i.

2. Language indifference: By **A.3.1** and **A.3.2**, in localities with a high percentage of bilinguals, and B as a common language of communication, bilinguals might feel linguistically satisfied. That is, they may feel that the actual α_i and the aspiration α^* were very close. Then they may overestimate the actual competitive power of B relative to A. In those circumstances, it might happen that bilinguals do not perceive any additional benefit from speaking B, nor any perceptible frustration when forced to use A; that is, both $m(\alpha_i) - n$ and $c(\alpha_i)$ will be nearly 0. If payoff differences between using either A or B are not perceived, bilinguals might develop linguistic indifference between the two official languages: $A \sim B$ (where $\sim$ denotes the binary relation of indifference).

Notice that we could have introduced *language B loyalty.* But this would just add more complexity without relevant changes, since loyalty would not change the negative slope of $m(\alpha_i)$ and $c(\alpha_i)$. It could only make them flatter.

4.3 Strategies

The pure strategies of the LUG, R and H, describe two frequent behaviours observed in minority bilingual populations and, therefore, in the sample population from which the data, used in the present work, are obtained. Thus, real-life bilingual behaviours are the

inspiration for the strategies H and R of the LUG. Strategy H represents (or has as a *prototype*) a conventional behaviour with respect to the use of B; i.e. H would describe the mainstream of the bilingual community. It would be those bilinguals who when meeting an unknown interlocutor would consider not polite and disrespectful to start the conversation in the minority language B (see [10]). It would also represent those bilinguals who, in high α_i contexts, tend to *code-switch* between B and A, and fall into the *language indifference,* $A \sim B$, mentioned in the previous section. It would include too those bilinguals who, in low α_i contexts, with low opportunities to speak the language, will make no efforts to avoid forgetting B.

Strategy R has as a *prototype* the militant of the minority language. Militants are frequently political and cultural activists. Usually, they know each other, and are well known by the rest of the community, as organizers of campaigns to promote the use of B or to change the laws when they constrain its use. They may also run the cultural clubs mentioned in the previous section, where bilinguals may meet and talk in B. Militants behave as *bilingual hunters.* If the militant has imperfect information about the interlocutor's linguistic type (bilingual or monolingual), then she, as a speaker or as a respondent, will use B or send signals of wanting to use B forcing the interlocutor to reveal her linguistic type. Militants of Basque are gathered around *Euskaraldia* [4]; see also [11]. For militants of Welsh, see [12] on the *Welsh Language Society*).

5 The LUG's Matrix of Expected Payoffs and the Replicator Equation

We assume that the LUG is played at each locality i of the country where B is spoken. We choose the year of the census or the sociolinguistic survey, and take as given the proportion of bilinguals reported for that year, α_i and the total number of bilinguals N_i of the locality. Linguistic preference (**A.3**) satisfaction induces a continuous play of the LUG, which in turn produces changes in the language use population state, and in the payoffs to the pure strategies. Hence we will have a language use dynamics in continuous time. We model this dynamics with the *one-population replicator dynamics* attached to the LUG. The replicators are the pure strategies R and H. At any moment t of time in a given locality i of the country under consideration, there is a *language use population state* $(N_{iR}(t)/N_i, N_{iH}(t)/N_i)$, where $N_{ij}(t)/N_i$ represents the share of bilinguals of locality i playing pure strategy j at t ($j = R, H$).

Let α_i be the proportion of bilinguals in locality i reported in the chosen year of the survey. From Fig 1 in the main text, describing the LUG, the bilinguals expect a payoff according to the pure strategy chosen and the realized state of nature. By assumption **A.2,** bilinguals know the local proportions α_i. Therefore, their *expectations* are based on that knowledge. Hence, they assign probabilities, α_i and $1 - \alpha_i$, to the *Bilingual* and *Monolingual* states, respectively. Then if the bilingual chooses strategy R and the realized state is *Bilingual*, she expects to get $m(\alpha_i)$ with probability α_i (no matter the strategy chosen by the bilingual interlocutor); and if the *Monolingual* state is realized she expects to get $n - c(\alpha_i)$ with

probability $1-\alpha_i$. This case is represented by the components a_{11}, b_{11}, a_{12} and b_{21} of the expected payoff matrix shown below. If the chosen strategy is H, and the realized state is *Bilingual* she expects to get $m(\alpha_i)$ with probability α_i if the bilingual interlocutor plays R; and if the *Monolingual* state is realized she expects to get n with probability $1-\alpha_i$. This case is represented by the components a_{21} and b_{12}. Finally, if both bilinguals choose to play H, they expect to get n for sure, as shown by the components a_{22} and b_{22}. Thus, the *expected payoffs* are represented in the following matrix:

	**R**	**H**
**R**	a_{11}, b_{11}	a_{12}, b_{12}
**H**	a_{21} , b_{21}	a_{22}, b_{22}

Where $a_{11}=b_{11}=a_{12}=b_{21}=\alpha_i(m(\alpha_i)-n)-c(\alpha_i)(1-\alpha_i)$, $a_{21}=b_{12}=\alpha_i(m(\alpha_i)-n)$, and $a_{22}=b_{22}=0$. Note that n has been deleted since it appears in all cells. By **A.3**, and a given $\alpha_i\in(0,\alpha^*)$, $\alpha_i(m(\alpha_i)-n)-c(\alpha_i)(1-\alpha_i)>0$ and $\alpha_i(m(\alpha_i)-n)>0$. This is a symmetric matrix with the structure of the well known so-called 'Hawk-Dove Game'. Let $p_i=N_{iR}(t)/N_i$ be the proportion of bilinguals of locality i who play strategy R at time t. The matrix $\mathbf{A}$ denotes the expected payoffs to the bilingual player in the row position (note that, by symmetry, the transpose of $\mathbf{A}$ denote the expected payoffs to column player):

$$\mathbf{A}=\begin{bmatrix} a_{11}, & a_{12} \\ a_{21}, & a_{22} \end{bmatrix}$$

Since the LUG is played continuously by pairwise random matches, we may assume that the minority language use population state, $\mathbf{p}_i(\mathbf{t})=(p_i(t),1-p_i(t))=(N_{iR}(t)/N_i,N_{iH}(t)/N_i)$, evolves as a differentiable function of time t (from now on, we delete t from the notation). The rate of increase of the share of bilinguals playing R, $\dot{p}_i/p_i$, depends on the expected payoffs to strategy R, $(\mathbf{A}\mathbf{p}_i)_R$, relative to the average expected payoffs, $\mathbf{p}_i^{\mathbf{T}}\mathbf{A}\mathbf{p}_i$. Thus, the *one-population replicator dynamics* associated to the LUG is:

$$\dot{p}_i/p_i=(\mathbf{A}\mathbf{p}_i)_R-\mathbf{p}_i^{\mathbf{T}}\mathbf{A}\mathbf{p}_i$$

Therefore,

$$\dot{p}_i=p_i(1-p_i)[\alpha_i(m(\alpha_i)-n)(1-p_i)-c(\alpha_i)(1-\alpha_i)]$$

is the differential equation for the evolution of the share of bilinguals playing pure strategy R who will use language B in the matchings with other bilinguals. The share of bilinguals who play H, will evolve in the opposite direction, so there is no need to indicate the differential equation $1-\dot{p}_i$. The rest points for the equation are $p_i=1$, $p_i=0$, and the interior evolutionary stable strategy (ESS) equilibrium $p_i^*=p^*(\alpha_i)$, obtained from $[\alpha_i(m(\alpha_i)-n)(1-p_i)-c(\alpha_i)(1-\alpha_i)]=0$.

$$p_i^*=p^*(\alpha_i)=1-\frac{(1-\alpha_i)c(\alpha_i)}{\alpha_i(m(\alpha_i)-n)} \tag{1}$$

Note that p_i^* is a global attractor for all $p_i \in (0,1)$: suppose all p_i such that $0 < p_i < p_i^*$. Then $p_i < 1 - \frac{(1-\alpha_i)c(\alpha_i)}{\alpha_i(m(\alpha_i)-n)}$ and so $\alpha_i(m(\alpha_i) - n)(1 - p_i) > c(\alpha_i)(1 - \alpha_i)$. Hence, $\dot{p_i} > 0$ which would lead p_i toward p_i^*. If $1 > p_i > p_i^*$, we can see, using the same procedure, that p_i decreases toward p_i^*. Thus, $p_i = 1$, $p_i = 0$ are unstable, and p_i^* is an evolutionary stable strategy (ESS) Nash equilibrium.

Remark 1: The equilibria of the LUG, in the framework of pairwise strategic interactions represented as a symmetric two player game, require the following assumptions:.

1. The player role or position in the game does not condition the choice of action. That is, both the row player (or player I) and the column player (or player II) will choose freely either R or H.

2. Who is the speaker (or who initiates the conversation), and who is the respondent is not conditioned by the player position nor by the choice of strategy. In particular, the row (column) player is not necessarily the speaker (respondent).

5.1 The Evolutionary Stable Equilibrium of the LUG as a Linguistic Convention

The ESS equilibrium, p_i^*, viewed as a *linguistic convention*, partitions the bilingual population of each locality, N_i, in two groups: the group $N_i p_i^* = N_{iR}^*$ consists of all the bilinguals of locality i who play strategy R in equilibrium; and $N_i(1 - p_i^*) = N_{iH}^*$, consists of all the bilinguals who play strategy H in equilibrium. Any member of N_{iR}^* will speak in B whenever she meets a bilingual (recall that strategy R discovers who in a matching is bilingual or monolingual). However, when a bilingual of the group N_{iH}^* meets another bilingual of the same group, they will speak in A. A bilingual of this latter group will use B only when she encounters a bilingual of the N_{iR}^* group.

As any other convention , the linguistic one, defined by p_i^*, will become a self-enforcing mechanism of (language) coordination [13], hard to remove, which could extend to domains where information is not private, such as family, friends. The Secretary of Linguistic Policy for Basque made the following statement in commenting the data of the *V Sociolinguistic Survey*: "In the last twenty years the use of Basque in the family has decreased one per cent." [14].

Note that, given α_i, when the ESS reaches values in the interval $p_i^* \in (0, 0.293)$, then the matching probability between bilinguals who will shift to A will be higher than the matching probability of bilinguals who will use B; that is, $(1 - p_i^*)(1 - p_i^*) > p_i^* \times p_i^* + p_i^*(1 - p_i^*) + (1 - p_i^*)p_i^*$.

6 Models of Expected Language B Use

The statistical *predictive use of B* models in the main text are based on the models of *expected use* of B which we describe in the following. The models of expected use are based

on the ESS equilibrium function $p_i^* = p^*(\alpha_i) = 1 - \frac{(1-\alpha_i)c(\alpha_i)}{\alpha_i(m(\alpha_i)-n)}$, obtained from (1), where α_i is the given bilingual proportion of locality $i \in$ {all localities quoted in the survey year of the country under consideration}. To calculate the expected percentage of conversations in language B at locality i, we should note that, by the LUG model, a militant of the B language (i.e. the prototype of an R player) must participate in each conversation. Therefore, we need to calculate the percentage of matches with militants involved. We only need two interlocutors to determine the language. Additional people, either will adapt or not join the conversation. We know from combinatorics that to calculate the frequencies of composite events, we need to take into account the order; that is, both (R *player* + H *player*) and (H *player* + R *player*) have to be counted. To these two events, one must add the frequency of (R *player* + R *player*). Since the frequency of an R player is $\alpha_i p_i^*$ and that of an H player is $\alpha_i(1-p_i^*)$, we get:

$$\alpha_i p_i^* \times \alpha_i(1-p_i^*) + \alpha_i(1-p_i^*) \times \alpha_i p_i^* + \alpha_i p_i^* \times \alpha_i p_i^* = 2\alpha_i^2 p_i^* - \alpha_i^2 p_i^{*2} \quad (2)$$

Note that (2) implicitly assumes that the observed frequencies correspond to the percentage of conversations in B resulting from random matches. However, one would realistically expect that, in the sample population of each locality, the use of B in the streets would occur both in random and non-random matches. The inclusion of the latter type of matches will (most likely) increase the use of B because of the bilinguals' language preferences, in particular, militants' preferences. Moreover, militants are more likely (i.e., more frequently) to know or recognize each other. This suggests that (2) will experience two kind of changes due to non-random matches. First, the percentage of conversations in B may increase as a whole; and second, for the given reasons, the fraction of matches with two militants involved will increase. These changes lead us to the following model of expected street use of B.

Expected Street Use (KE) : Based on the ESS equilibrium function $p_i^* = p(\alpha_i)$ (1), for a given α_i, we propose the following expected street use of B in locality i:

$$E[KE|\alpha_i, p_i^*] = c_1\left(2\alpha_i^2 p_i^* - c_2\alpha_i^2 p_i^{*2}\right) . \quad (3)$$

with unknown constants $c_1 > 0$ and $c_2 \leq 1$. When $c_1 = c_2 = 1$, we will have the same percentage of conversations in B as in the unrealistic situation of only taking into account random matches. One may argue that $c_1 \approx 1$, assuming that the change in (2) is mainly due to the increased matches between militants. For that reason, c_2 is likely to be much smaller than 1. After trying different values, we found an excellent data fit when $c_2 = 0$ in the empirical predictive street use of the main text.

Expected Daily Use (DU):

$$E[DU|\alpha_i, p_i^*] = c_0\alpha_i p_i^* . \quad (4)$$

for an unknown positive constant c_0. For modelling the expected daily use, it is assumed that almost all individuals who play strategy R will answer (in the census or survey) that

they use B every day, whereas almost all individuals playing strategy H will answer that they do not. This would actually assume that $c_0 \approx 1$. However, in the main text we build the empirical daily use model, and show that we do not need to assume $c_0 \approx 1$.

7 Econometric Specification of the Models of Predictive Use of Basque, Irish and Welsh

The predictive models will test our LUG model along the observed data of street use of Basque KE_i, and local daily use of Irish and Welsh, DU_i. To build the empirical versions of $PKE(\alpha_i)$ and $PDU(\alpha_i)$, we need to specify the function $p^*(\alpha_i)$. We start by specifying $m(\alpha_i)$ and $c(\alpha_i)$ as simple functions satisfying **A.3**. Let us suppose that $m(\alpha_i)$ is a simple decreasing function of the type $\frac{K}{\alpha_i}$, where $K > 0$ is a constant. For any $\alpha_i < \alpha^* < 1$, where α^* is the country's aspiration, we have $m(\alpha_i) > n$, such that the bilingual speaker of locality i gets a positive profit whenever she coordinates in B. Profits would be zero in the hypothetical case where $\alpha^* < 1$ is reached; that is, at α^*, $m(\alpha^*) = \frac{K}{\alpha^*} = n$. Then for $\alpha_i < \alpha^*$, and a given value of $n > 0$, we get $K = \alpha^* n < n$, and so the weighted profit $b(\alpha_i) = (m(\alpha_i) - n)\frac{\alpha_i}{(1-\alpha_i)}$ is a decreasing function in α_i.

By assumption, **A.3**, for any $\alpha_i < \alpha^*$, the frustration cost function is strictly smaller than the weighted benefit function:

$$c(\alpha_i) < (m(\alpha_i) - n)\frac{\alpha_i}{1-\alpha_i}$$

Then

$$c(\alpha_i) = (m(\alpha_i) - n)\frac{\alpha_i}{(1-\alpha_i)} - \tilde{b}(\alpha_i) \tag{5}$$

for some $\tilde{b}(\alpha_i) > 0$ denoting the net benefit. Inserting equation (5) in (1) we obtain

$$p^*(\alpha_i) = \frac{\alpha_i(m(\alpha_i) - n) - c(\alpha_i)(1-\alpha_i)}{\alpha_i(m(\alpha_i) - n)} = \frac{\tilde{b}(\alpha_i)(1-\alpha_i)}{\alpha_i(m(\alpha_i) - n)} ,$$

and substituting $m(\alpha_i) = \frac{K}{\alpha_i}$ gives

$$p^*(\alpha_i) = \frac{\tilde{b}(\alpha_i)(1-\alpha_i)}{K - n\alpha_i} . \tag{6}$$

Equation (6) shows how the ESS equilibrium proportion of the bilingual population playing R changes with α_i. The denominator is positive because $m(\alpha_i) = \frac{K}{\alpha_i} > n$ for all $0 < \alpha_i < \alpha^*$. Hence $p_i^* = p^*(\alpha_i) > 0$, and it is increasing in α if $\tilde{b}(\alpha_i)$ is not decreasing faster than $\frac{1-\alpha_i}{\alpha_i(m(\alpha_i)-n)}$ is increasing. Note that the first derivative of $g(\alpha_i) = \frac{1-\alpha_i}{\alpha(m(\alpha_i)-n)}$ with respect to α_i is $g'(\alpha_i) = \frac{n-K}{(K-n\alpha_i)^2}$. Hence, for $\alpha_i < \alpha^* < 1$, $g'(\alpha_i) > 0$ since $K = \alpha^* n < n$. So one would assume that the net benefit function $\tilde{b}(\alpha_i)$ has a well defined first derivative. Finally, let $\check{b} := \tilde{b}/n$ denote the relative net benefit (recall, $n > 0$ is constant). Substituting $K = n\alpha^*$ in the denominator of (6), we get

$$p^*(\alpha_i) = \frac{\check{b}(\alpha_i)(1-\alpha_i)}{(\alpha^* - \alpha_i)} . \tag{7}$$

Since $p^*(\alpha_i) \in (0,1)$, we have $\check{b}(\alpha_i)(1-\alpha_i) < (\alpha^* - \alpha_i)$, where $1 > \alpha^* > \alpha_i > 0$, and hence $0 < \check{b}(\alpha_i) < 1$. As said above, the *aspiration function* $S(\alpha_i)$ arranges all local aspirations inside a similarity interval, reflecting the agreement among the local aspirations, α_i^*, and the country's aspiration α^*. That is, for all α_i, $S(\alpha_i) = \alpha_i^* \simeq \alpha^*$, with $0 < \alpha_i < \alpha^* < 1$ (where $\simeq$ denotes similarity). Then we may specify $\alpha^* \simeq \alpha_i^* = \alpha_i^{\beta_3}$, and capture **A.3.3** by specifying $\check{b}(\alpha_i)$ as $\check{b}(\alpha_i) = \beta_1(\alpha_i^{\beta_3} - \alpha_i)^{\beta_2}$, for unknown β_1, β_2, β_3; for the model to make sense, we work with the restrictions $\beta_1 > 0$ and $0 < \beta_3 < 1$. Then (7) is converted into

$$p^*(\alpha_i) = \beta_1(1-\alpha_i)(\alpha_i^{\beta_3} - \alpha_i)^{\beta_2 - 1} . \tag{8}$$

Using (8), we substitute $p^*(\alpha_i)$ in the $PKE(\alpha_i)$ model, where it is assumed $c_2 = 0$, and in the $PDU(\alpha_i)$ model of predicted use of B in equilibrium:

$$PKE(\alpha_i) = 2c_1\beta_1\alpha_i^2(1-\alpha_i)(\alpha^{\beta_3} - \alpha_i)^{\beta_2 - 1} = \widetilde{\beta}_1\alpha_i^2(1-\alpha_i)(\alpha^{\beta_3} - \alpha_i)^{\beta_2 - 1} \tag{9}$$

$$PDU(\alpha_i) = c_0\beta_1\alpha_i(1-\alpha_i)(\alpha_i^{\beta_3} - \alpha_i)^{\beta_2 - 1} = \breve{\beta}_1\alpha_i(1-\alpha_i)(\alpha_i^{\beta_3} - \alpha_i)^{\beta_2 - 1} \tag{10}$$

These are the models we will study empirically for Basque, Irish and Welsh.

Remark: It should be clear that for each (census or linguistic survey) year and language, the predictive models have three parameters: either $\widetilde{\beta}_1$ or $\breve{\beta}_1$, β_2 and β_3.

As it happens with all models, some assumptions of the LUG could be, in practice, violated. For instance, when in some matches interlocutors are fully informed of the linguistic type of each other. This is the reason why in the empirical analysis error terms are added. More specifically, in order to estimate the models from the samples $\{KE_{cti}, \alpha_{cti}\}_{i=1}^{n_{ct}}$, $\{DU_{cti}, \alpha_{cti}\}_{i=1}^{n_{ct}}$ for language sample c in year t, one might consider either

$$KE_{cti} = PKE_{cti} + \varepsilon_{cti} \ , \quad DU_{cti} = PDU_{cti} + \epsilon_{cti} \ , \tag{11}$$

(where ε_{cti} and ϵ_{cti} are mean zero error terms) or, as a model with multiplicative structure,

$$\log(KE_{cti}) = \log(PKE_{cti}) + \tilde{\varepsilon}_{cti} \ , \quad \log(DU_{cti}) = \log(PDU_{cti}) + \tilde{\epsilon}_{cti} \ . \tag{12}$$

This is done by least squares under the constraints that $\widetilde{\beta}_1 > 0$, $\breve{\beta}_1 > 0$ and $0 < \beta_3 < 1$. Note that (11) and (12) require different assumptions on the (random) deviations from the mean. They are, however, not testable, so that we have no particular a priori preference. While the general findings are quite similar resulting from one or the other estimation strategy, predicting the language use from the logarithmic version (and consequently $\widehat{\log(KE)}$ or $\widehat{\log(DU)}$) is somewhat more complex since one has to correct for the - in our case heteroscedastic - error dispersion because $E[\log(KE)|\alpha_i] < \log E[KE|\alpha_i]$. The same holds for DU. Also, the data fits when looking at the untransformed data did not look very convincing. We therefore give the least square estimates resulting from model (8). All estimates

are shown, together with nonparametric fits of KE (DU) on α_i using local linear estimators and local bandwidths with Epanechnikov kernel. Specifically, we used the command `locfit` of the R-package 'locfit' with degree 1 and nearest neighbor fraction set to 0.25 for Basque and Welsh, but the 0.7 for Irish due to the extremely asymmetric distribution. These settings were also used for the test statistics.

8 The Predictive Equilibrium Functions and Parameter Estimates

In Tables S1-S3 are given the estimates of $\widetilde{\beta}_1$, $\breve{\beta}_1$, β_2 and β_3, toghether with the number of observations per year, their bootstrap standard errors and 95% bootstrap confidence intervals. Note that the parameter estimates are highly correlated to each other such that their individual confidence interval are of little practical interest. Further, recall that our estimates of $\widetilde{\beta}_1$ and $\breve{\beta}_1$ are even a nonseparable (and therefore hardly interpretable) composition of model parameters. However, this is not a problem fot our investigation. Recall that we have a behavioural game model, the LUG, whose evolutionary stable equilibrium predicts, in each locality i where B is spoken, the proportion of bilinguals who shift to language A. The purpose of the empirical study is the validation of the LUG model, not the interpretation of numerical outcomes of any parameter. Hence, instead of looking at the confidence intervals of the parameter estimates, we should concentrate on the confidence intervals of the empirical predictive equilibrium functions $PKE(\alpha_i)$ and $PDU(\alpha_i)$, which are the empirical representation of our LUG model. We want to know whether the predictions of $PKE(\alpha_i)$ and $PDU(\alpha_i)$ fit well the observations of minority language use.

While the confidence bands of the functions are very narrow, the 95% confidence intervals for the parameter estimates are quite wide due to extremely high correlations (not shown) between the parameter estimates. These, like standard errors and the *p-values* of our test, are estimated by wild bootstrap. In Figs. S1-S3, the thick solid line is the model-based estimate with confidence bands indicated by thin solid lines. The dashed lines are the nonparametric (i.e., model-free) data fits, and the grey circles indicate the recorded observations. To see better the curvature, we have also plotted a 45^0 line, and a horizontal line at $\alpha_i = 0$. The functions are estimated from different samples taken from the language use data sources. For Basque we have data of α_i and street use, KE_i, from between 53 to 175 municipalities for the years 1993, 1997, 2001,2006, 2011 and 2016 (source: *Soziolinguistika Klusterra*). For Irish, we have data from about 180 so-called 'local electoral areas' obtained from different censuses. Note that for Irish we mainly refer to 2006 and 2011 because it is only since 2006 that there is a clear definition of daily use, DU_i, outside the educational system (we are uncertain that the DU_i of 2002 included the use of Irish in the schools or not). For Welsh DU_i data are from 22 local authorities (2005 and 2014) (source: [2]). The sources of data are given in section 1 of the present SI.

The $PDU(\alpha_i)$ for Welsh is almost parallel to the 45° line. When α_i is above 0.6, so bilinguals are a clear majority, then the function immediately shifts to the 45° line, and has the same

properties as those of Basque. For Irish we see a clear difference as compared to Basque and Welsh. The latter two have a more intensive use than Irish.

All model-based estimates come very close to the nonparametric (i.e. model-free) data fits, excepts maybe for extreme values and outliers. In particular, most of the time the confidence bands include the nonparametric fit. This clearly supports the LUG model.

Each empirical equilibrium model, the $PKE(\alpha_i)$ for Basque and the $PDU(\alpha_i)$ for Irish and Welsh, gives rise to an strictly increasing and convex relation between the local proportion of bilinguals, α_i, and the predicted local use of B in equilibrium. It could be argued that intuition might suggest that the predictive functions should be increasing and convex, However, these two properties alone cannot explain the variation observed in the data. There is, still, plenty of space for model mis-specification.

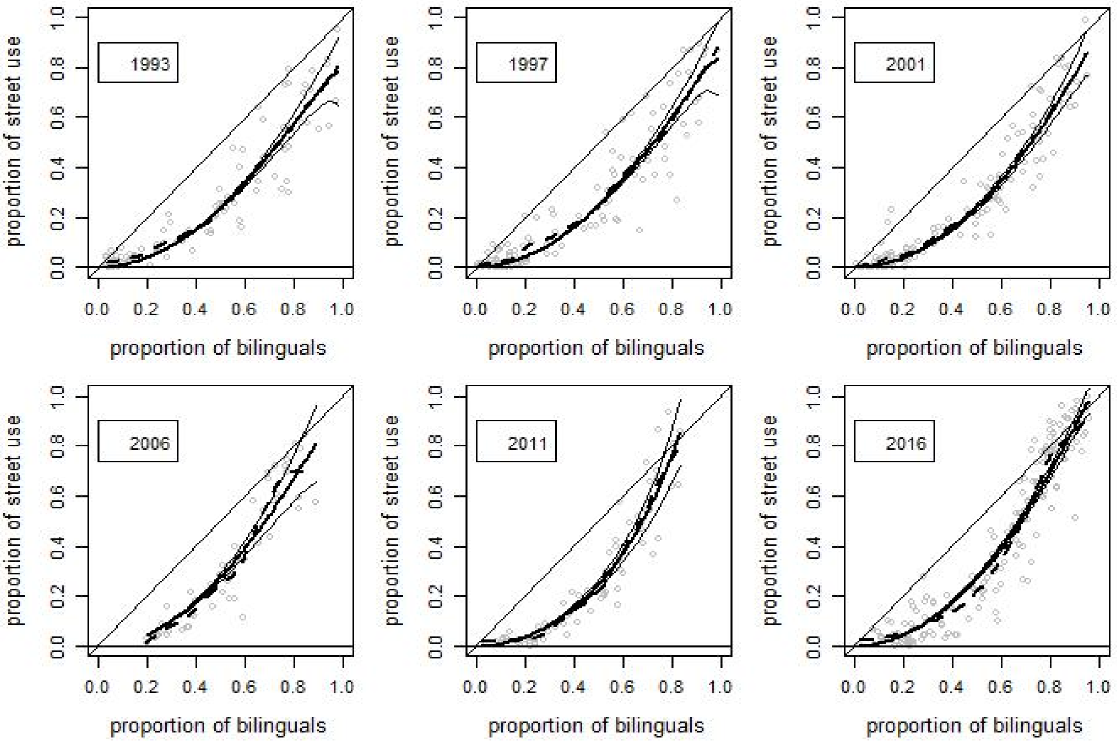

Figure 1: $PKE(\alpha_i)$ **for Basque**: Solid lines. Thick line is the estimate, thin lines give the 95% confidence bands. The dashed lines are nonparametric kernel regression fits.

8.1 Confidence Bands

We continue the empirical study with another formal test. We say 'another' since the provision of confidence bands is equivalent to a particular statistical test. More specifically, we apply the nonparametric specification test of Härdle and Mammen [15] which takes as test statistic the Euclidean distance between the smoothed version of the model-based parametric fit and the nonparametric fit. The 'smoothed version' means that the parametric

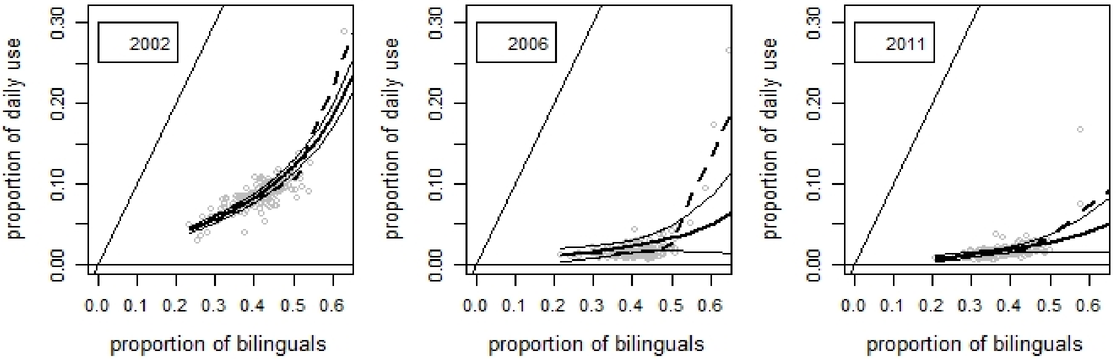

Figure 2: $PDU(\alpha_i)$ **for Irish**: Solid lines. Thick line is the estimate, thin lines give the 95% confidence bands. The dashed lines are nonparametric kernel regression fits.

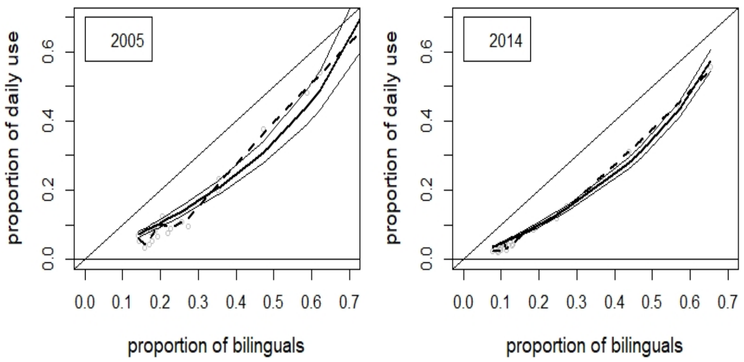

Figure 3: $PDU(\alpha_i)$ **for Welsh:** Solid lines. Thick line is the estimate, tin lines give the 95% confidence bands. The dashed lines are nonparametric kernel regression fits.

Table 1: Parameter estimates (with standard deviations in parenthesis) and 95% bootstrap confidence intervals (C.I.) for the Basque municipals; *obs.* indicates number of observations.

year	1993	1997	2001	2006	2011	2016
$\tilde{\beta}_1$	.915 (.044)	.890 (.027)	.946 (.024)	1.07 (.026)	.849 (.036)	1.10 (.024)
C.I.	[.880;.944]	[.857;.929]	[.918;1.02]	[1.03;1.13]	[.776;.934]	[1.09;1.17]
β_2	.041 (.018)	.030 (.015)	.003 (.014)	.039 (.036)	-.191 (.031)	.008 (.009)
C.I.	[.008;.077]	[.000;.060]	[-.019;.035]	[-.021;.116]	[-.233;-.116]	[.000;.033]
β_3	.044 (.040)	.089 (.024)	.011 (.011)	.032 (.023)	.013 (.023)	.001 (.005)
C.I.	[.019;.074]	[.055;.116]	[.000;.036]	[.001;.096]	[.001;.088]	[.000;.015]
obs.	96	116	127	53	74	175

Table 2: Parameter estimates (with standard deviations in parenthesis) and 95% bootstrap confidence intervals (C.I.) for the Irish local election areas; *obs.* indicates number of observations.

year	2002	2006	2011
$\breve{\beta}_1$	.043 (.012)	.007 (.379)	.020 (.139)
C.I.	[.042;.085]	[.007;1.59]	[.016;.535]
β_2	-1.31 (.083)	-1.42 (1.26)	-1.02 (.801)
C.I.	[-1.33;-1.04]	[-1.509;3.06]	[-1.12;2.22]
β_3	.237 (.044)	.317 (.113)	.100 (.099)
C.I.	[.092;.245]	[.019;.547]	[.013;.531]
obs.	180	180	200

Table 3: Parameter estimates (with standard deviations in parenthesis) and 95% bootstrap confidence intervals (C.I.) for the Welsh local authorities.

	2004-2006		2013-2015	
	estimate (s.e.)	95% C.I.	estimate (s.e.)	95% C.I.
$\breve{\beta}_1$	.461 (.046)	[.349;.522]	.429 (.020)	[.390;.474]
β_2	-.538 (.050)	[-.613;-.414]	-.667 (.026)	[-.693;-.585]
β_3	.004 (.035)	[.0004;..095]	.004 (.008)	[.0002;.027]
obs.	22		22	

fit gets convoluted with the same kernel and bandwidth we used for the nonparametric fit to avoid rejecting our hypothesis simply because of a smoothing bias inherited by the nonparametric fit. Then the bootstrap p-values are: for Basque 0.728 for 1993, 0.408 for 1997, 0.559 for 2001, 0.054 for 2006, 0.479 for 2011, and 0.003 for 2016. For Irish 0.055 for 2002, 0.032 for 2006, and 0.095 for 2011; and for Welsh we get a p-value of 0.075 for 2005 and 0.080 for 2014. This, together with the graphs, indicate that the model adapts reasonably well to the observed data. The reason for the low p-values in some years, that can also be detected in the pictures, is due to the α_i values for which the nonparametric line is far outside the confidence bands.

To calculate the p-values, uniform confidence bands, standard errors and confidence intervals, we generated 1000 bootstrap samples $\{Y_i^*, \alpha_i\}_{i=1}^n$ where $Y_i^* := \widehat{Y}_i + (Y_i - \widehat{Y}_i) \cdot \varepsilon_i$ with Y_i being KE_i (or DU_i), $\widehat{Y}_i$ our estimated prediction model, and ε_i randomly drawn from a standard normal. We also checked with centred and normalized chi-square to guarantee non-negative responses [15], and a two-point distribution to account better for potential asymmetries [16]. We present the version with standard normal ε_i as these seem to calibrate best nonparametric test for moderate sample sizes [17]. Then, for example the uniform

confidence bands for PKE were constructed in the following way. Define

$$T := \sup_{\alpha\in(0,1)} |PKE(\alpha_i) - \widehat{PKE}(\alpha_i)|/\sigma_y(\alpha_i)$$

with $\sigma_y^2(a) = Var[\widehat{PKE}(a)]$. It can be shown that its bootstrap analogue

$$T^* := \sup_{\alpha\in(0,1)} |PKE^*(\alpha_i) - \widehat{PKE}^*(\alpha_i)|/\sigma_y^*(\alpha_i)$$

is converging in distribution to T. Consequently, from our bootstrap samples we can obtain any quantile q_T of T. The formula $\widehat{PKE}(\alpha_i) \pm q_T^* \sigma_y^*(\alpha_i)$ provides us with uniform confidence bands at any wanted quantile. The confidence bands (as the confidence intervals) can be asymmetric because they are calculated by this bootstrap method, and therefore reflect the asymmetry of the distributions of our estimators.

These uniform confidence bands are very useful as the nonparametric test simply tells us whether the theory model has a low p-value or not; it does not tell us where a potential problem is. In our case, the bands provide us with the information that there is not a general, systematic deviation of our theory model from the nonparametric fit. In fact, apart from outlier problems on the right hand side of the Irish data, the low p-values for Basque in 2016 and Irish in 2006 are due to an 'elbow' of the real data (respectively the nonparametric fit) at around $\alpha_i = 0.5$, i.e. when bilinguals start to become a majority.

We can conclude that we could not find any empirical evidence against our theory based model.

8.2 Nonparametric Estimation procedure

For ease of notation we always use KE_i as observed response variable; for DU_i, the methodology works exactly the same way. Given a sample $\{\alpha_i, KE_i\}_{i=1}^n$ one wants to estimate the conditional expectation $E[KE_i|\alpha_i] = g(\alpha_i)$ under the assumption that $g(\cdot)$ is a smooth function having second order Lipschitz continuous derivatives. The errors $v = KE_i - g(\alpha_i)$ have finite variance. One may add some conditions on the distribution of α_i if one wants to calculate the statistical properties of the now described estimator: For a weight or kernel function $K(\cdot)$ for which we chose the Epanechnikov kernel $K(u) = 0.75 \cdot (1-u^2)_+$ (the subindex $+$ indicates that the function is set to zero if $1-u^2$ is negative) and bandwidth h_x we take

$$\widehat{g(x)} = \underset{g,g_1}{argmin} \sum_{j=1}^{n} (KE_j - g - g_1 \cdot (\alpha_j - x))^2 K(\frac{\alpha_j - x}{h_x}) \tag{13}$$

as an estimate for $g(x)$. This is the local linear kernel estimator. Letting x run over the range of α (over all sample observations α_i) we can draw than the function estimate of $g(\cdot)$ which is compared with our model for $PKE(\alpha_i)$.

9 References

[1]. Altuna O, Basurto A (2013) *A Guide to Language Use Observation. Survey Methods* (Soziolinguistika Klusterra).

[2]. Jones, HM (2012). *A Statistical Overview of the Welsh Language*, The Welsh Language Board. Cardiff.

[3]. Information about the *Welsh Language Commissioner* is available at https://www.welshlanguagecommiss

[4]. Information about *Euskaraldia* is available at https://euskaraldia.eus/es/

[5]. Kahneman D, Tversky A (1979) Prospect theory: An analysis of decision under risk. *Econometrica* 47(2): 263-289.

[6]. Landry R, Bourhis RY (1997) Linguistic landscape and ethnolinguistic vitality an empirical study. *Journal of Language and Social Psychology* 16 (1): 23–49

[7]. Rubinstein, A. *Modeling Bounded rationality.* The MIT press. Cambridge, Massachusetts

[8]. Begum G and Richter M (2015) An experiment on aspiration-based choice. *J Econ Behav Org* 119, 512–526.

[9]. Begum G, Richter M, Tsur M (2016) Aspiration-based choice theory. Working Paper.

[10]. Amorrortu, E,, Ortega, A., Idiazabal, I. and Barrena, A. (2009) *Actitudes y Prejuicios de los Castellanohablantes Hacia el Euskera.* Jaurlaritza- Basque Government: Vitoria-Gasteiz.

[11]. Urla, J. (2012) *Reclaiming Basque. Language, Nation, and Cultural Activism.* University of Nevada Press. Reno, Nevada.

[12]. Jenkins, G.H. and Williams, M.A. (2015) *Let's Do Our Best for the Ancient Tongue. The Welsh Language in the Twentieth Century.*University of Wales Press. Cardiff, U.K.

[13]. Young, H.P. (1995) The economics of convention. *J Econ Perspect* 10:105-122.

[14]. Baztarrika, P. (2014) Euskararen hazkundearen paradoxak. www.erabili.eus/zer_berri/muinetik/.

[15]. Härdle, W. and Mammen, E. (1993) Comparing Nonparametric Versus Parametric Regression Fits. *Ann Stat* 21(4): 1926–1947.

[16]. Pendakur, K., Scholz, M. and Sperlich, S. (2010) Semiparametric indirect utility and consumer demand. *Comput Stat Data Anal* 54: 2763–2775.

[17]. Sperlich, S. (2014) On the choice of regularization parameters in specification testing: a critical discussion. *Empirical Economics* 47(2), 427–450.
