# Supplementary material for: A behavioural model of minority language shift: Theory and empirical evidence
Source: PLoS One. 2021 Jun 4;16(6):e0252453. doi: 10.1371/journal.pone.0252453 (PMC8177434; doi:10.1371/journal.pone.0252453)
Supplement: Attachment — Submitted filename: Response to Reviewer 2.pdf [file pone.0252453.s003.pdf]

Answer to Reviewer #2:

We appreciate very much your comments. These are the answers to each of your comments:

1. "*My knowledge of game theory and statistics is not sufficient to judge the corresponding equations*"

Answer 1:

The provide the basic knowledge for a smoother reading of the paper, we have made some changes in the Intro, explaining step-by-step why game theory and evolutionary game theory are the right framework to study the bilinguals' language choice behaviour. Further, before describing the Language Use Game, we have introduced an example where the concepts of strict Nash equilibrium and Evolutionary Stable Equilibrium as an emerging Convention.

An example: The Rules of the Road Game

Let us consider the game of which side of the road to drive on, whether left side or right side (for more details, see Young (1996)). The game is played by two oncoming vehicles. Since drivers want to avoid accidents, they will coordinate on the same side of the road. Hence, this is a coordination game. Drivers have no incentives to deviate from coordinating on the same side because they are strictly better off than being involved in an accident. This is the basis for the notion of strict Nash equilibrium. Hence, this coordination game has two strict Nash equilibria (Left, Left) and (Right, Right). These equilibria are two rules or conventions. But which of the two equilibria will be chosen? which is the same as asking which of the two conventions, driving on the right or driving on the left, will be adopted by the society? To answer this question, we should view how the game evolves historically by the repetition of the interaction. We can think of the game being played by a large population. Each time a different pair of drivers will play the game and may remember how the interaction was solved. As the game is repeated over and over by many people, one particular way of solving the interactions, say (R, R), takes prominence in the population, driving out the other one, (L, L). This process of emerging the convention is by accumulation of precedent. In our view, this mechanism in which imitation plays an important role might explain the linguistic convention built by bilinguals in the present paper.

A convention may become established through a different mechanism. Initially, when traffic was scarce, some area of the country might adopt, by local custom, say, the left convention, while other areas of the country adopt

the right convention. As traffic grew in volume the evolution of those conventions will be affected by sudden changes in the law regulating the traffic at the national level. These changes will also consider the chosen conventions made by the neighboring countries. Finally, the society decides politically to establish one convention. These two mechanisms are not mutually exclusive. Note that in terms of welfare both conventions, (R, R) and (L, L), are equally good. What the established convention does is to solve the indeterminacy. That is, we may define a convention that has emerged historically as an equilibrium that everybody expects in interactions that have more than one equilibrium, as in the present game. In other words, expectations and behaviors are in equilibrium in the established convention. Hence, the convention that has emerged and has been adopted by the society, relative to the Rules of the Road Game, is an evolutionary stable strategy.

In the Language Use Game we show that bilinguals converge into an evolutionary stable equilibrium in which they do not all play the strategy that will coordinate them in speaking language B.

2. *"Some details:*

Why not show data points of recorded observations in the figures in the main text?"

Answer 2:

One could do that but then one has to reorganize the visual presentation of results. There are several reasons for our choice, the most important ones are the following:

To be concise, but as informative as possible, we thought it is more interesting to show for each linguistic area the development over years.

This makes it impossible to jointly show the data points inside Figure 2 (you cannot show the point clouds of all the years in one picture, because then you see nothing!). Instead, we show the boxplots of the observations. For the common reader, together with the estimates relating alpha to language use, they are actually more informative than 11 pictures of point clouds (2 for Welsh, 3 for Irish, and 6 for Basque).

3. *"Fig.2: I think the axis should read "fraction", not "%", same in figures in supp.info."*

Answer 3:

We depict in the horizontal axis the observational variable alpha, which, all along the text, is referred to as the proportion of bilinguals. When in the

text it is written down as $\alpha_i \in (0, 1)$, it denotes the proportion of bilinguals of municipality i. We do not think there is a problem here since the proportion of bilinguals, denoted %, and the fraction of bilinguals in the total population of a municipality are mathematically equivalent.

Finally: all typos are corrected.

Reference:

Young, H.P. (1996), The economics of convention. Journal of Economic Perspectives, Volume 10, No. 2:105–12
