# Supplementary material for: A behavioural model of minority language shift: Theory and empirical evidence
Source: PLoS One. 2021 Jun 4;16(6):e0252453. doi: 10.1371/journal.pone.0252453 (PMC8177434; doi:10.1371/journal.pone.0252453)
Supplement: Attachment — Submitted filename: Response to Reviewer 2.pdf [file pone.0252453.s004.pdf]

Response to Reviewer 2

Thank you for recognizing the value of our work, for your careful reading and your suggestions to further improve our paper, to make it accessible to a broader readership. We have revised the entire text accordingly but without marking any tiny change or twisting of words in blue. We are well aware that also this version may not be fully understand in all technical details, but we have tried very hard to relax the technical language without loosing the scientific rigorousness. In the following we answer to your comments (in a slightly modified order, just to link or merge related issues) point by point. We hope this new version addresses your concerns in a satisfactory way.

Comment 1:

"I still think that this is a highly interesting new approach to language shift touching on psychology and sociology, and that it certainly deserves publication. The way it is presented, however, is a real challenge to possible readers not familiar with game theory, and that could be the overwhelming majority. Being until now not familiar with game theory I tried to learn the concepts and to check whether the conclusions of the paper appear plausible. I have to confess that still I can not follow all steps."

Answer 1:

Thank you for your kind words. It is indeed a completely novel approach to minority language shift, making use of a behavioural game model, namely the Language Use Game (LUG). We therefore use a more formal (one may say mathematical) approach to language shift. In a formal analysis, there are certain rules and terminology to present the methodology and the empirical validation. In this sense we are looking for a compromise that allows us to comply with those rules but at the same time results in a paper accessible to a broader audience (as you suggest).

Certainly we are not the first in using mathematical tools to investigate this issue. In contrast to ours, (1)-(10) investigate language competition and shift by means of, at least, equally technical, mathematical models (i.e. often based on complex differential equations for diffusion model from theoretical physics). In fact, the quoted authors in this strand are mostly mathematicians and physicists. But some authors are either linguists or members of linguistic departments, e.g. (6) and (10). In our paper we really try (thanks to your criticism, outlined further below) to find a way to reach both communities. However, we are aware that it always will be a kind of 'compromise', and we hope for your understanding.

Having in mind readers who do not belong to the very technical strand, i.e. are not familiar with our methodology, we revised the entire text again, acknowledging your interest and efforts to get the message of our work through all the concepts and mathematical derivations. We believe that now have found an adequate mixture of rigorous technical parts and intuitive examples and explanations (or wording). In the present second revision we also introduced some additional clarifications.

Comment 2:

"On page 8 you write: The replicators are the pure strategies R and H." For non-specialists (e.g. me) at least a little surprising. How can...? But certainly correct (for game theorists), could you explain in detail?"

Answer 2:

Thanks for pointing this out; you will see that in this new version we added much more explanations or changed the wording to provide a more intuitive approach. The standard replicator dynamics, which is the one used in our work, presumes that bilingual individuals only play the pure strategies of language use: R and H. These strategies can be copied or replicated. That is, the choice of R by one bilingual can be adopted, by way of imitation, by other bilingual(s). Similarly, for H. The percentage of bilinguals playing a given strategy changes continuously. It can be expanded or contracted depending on the expected payoffs to the strategy relative to the average expected payoff of the system. This is what the replicator dynamics will tell us. At each moment of time, the replicator equation (see below why we say "equation", in singular) will give us information about the proportion of bilinguals playing R and the proportion of those playing H.

Comment 3:

SI 9: new term: "one-population replicator dynamics". Why "one-population"?

Answer 3:

Probably you are thinking of something more complicated, but we actually refer to something quite simple: the LUG is only played by the population of bilinguals because monolinguals cannot choose language. This is emphasized now a bit more. Consequently, the LUG is a one-population game, from which we obtain the one-population replicator dynamics.

Comment 4:

"I think that in particular Theorem 1 (page 9) could be presented in a

way which permits better access to non-specialists.

Some ideas:

• Realize that the terminology might be repulsive for linguists et al. Could you try not to shock non-specialists with terminology like: “standard replicator dynamics”, “replicator dynamics equation” in other place just called “replicator equation”, “evolutionary stable state strategy ESS”, “Nash equilibrium”, “global attractor”, most of which presented in just 3 lines on page 9? Of course, by searching in Wikipedia also non-specialists can learn what all that means, but will they do so? And I doubt that you really need all of this terminology: it could be mentioned in footnotes for specialists who otherwise might be frustrated to miss it.

• Structure the explanation of the most important Theorem 1, i.e. in particular allow more space for deriving the replicator equation for the derivative of p_i. Possibly without making extensive use of game-theory terminology.

I believe equ. (1) is correct, but have to confess that I was not able to convince myself.″

Answer 4:

1. Presentation of Theorem 1:

We see your point. In this spirit but also sticking to what we said before (looking for a ’compromise’) we revised that part. In particular, **see the presentation of the theorem in the Introduction** and the discussions right before and after the Theorem.

We believe that the reader does not need to be a game theory specialist to accept that conventions (of any nature) are quite stable, and even hard to change. Recall that the **Example** on the admittedly somewhat trivial ’road game’ introduces the main game concepts needed, see also **Remark 3** for a an intuitive understanding of the theorem. It basically says that the mentioned equilibrium of the LUG is a linguistic convention explaining why a proportion of bilinguals may shift to the majoritarian language A.

Let us try to explain now how to obtain equation (1), our equilibrium p_i^*.

2. The derivation of p_i^* and its stability:

We have added this in the SI, in order to see how one could proceed to obtain equation (1). It is probably simpler than you might believe: We need to know the points where the replicator dynamics equation stops moving. Those points are called the rest points of the equation. Consider the points $p_i = 0$ and $p_i = 1$. If we substitute these values of p_i on the right-hand side (of the equality) of the replicator equation, then the dynamics stops;

i.e., $\overset{\bullet}{p}_i = 0$. Hence, $p_i = 0$ and $p_i = 1$ are rest points. Now, let us take the expression inside brackets [...]. If we equalize this expression to 0, then $\overset{\bullet}{p}_i = 0$ as well. Specifically, consider

$$\alpha_i(m(\alpha_i) - n)(1 - p_i) - c(\alpha_i)(1 - \alpha_i) = 0$$

Solving this equation for p_i , you would obtain the equilibrium $p_i^* = 1 - \frac{(1-\alpha_i)c(\alpha_i)}{\alpha_i(m(\alpha_i)-n)}$ at which $\overset{\bullet}{p}_i = 0$. So p_i^* is a rest point. Is it stable? Consider any any p_i such that $0 < p_i < p_i^*$. Then $p_i < 1 - \frac{(1-\alpha_i)c(\alpha_i)}{\alpha_i(m(\alpha_i)-n)}$ and so $\alpha_i(m(\alpha_i) - n)(1 - p_i) > c(\alpha_i)(1 - \alpha_i)$. Hence, $\overset{\bullet}{p}_i > 0$, which would lead p_i toward p_i^*. For any p_i, such that $1 > p_i > p_i^*$, using the same procedure, we can see that $\overset{\bullet}{p}_i < 0$ decreases toward p_i^*. Hence, dynamically p_i^* is stable and a global attractor in $(0, 1)$ and both $p_i = 0$ and $p_i = 1$ are unstable rest points. As said, **we have added this explanation now in the SI**.

We hope to have answered your doubts in an understandable way.

Comment 5 with Answer:

Typing errors and alike: We are very thankful for these comments and have tried to address them all, except point 4 (we are aware that different communities unfortunately sometimes use different notations). Note that $g'(\alpha_i)$ denotes the derivative of the function $g(\alpha_i)$ with respect to α_i.

Comment 6:

Terminology:

1. Please define “private information”.

2. Is there a difference between “payoff” and “benefit”? On SI p.13 you even sometimes change from benefit to profit, that appears confusing. And how about “utility”, e.g. “perceptible utility gain” (SI p.7, third line from bottom) - what is difference from payoff or benefit? My confusion continues on SI p.8 where new term “preference intensity” appears.

3. SI 9: new term: “one-population replicator dynamics”. Why “one-population”?

4. SI p.10: “Hawk-Dove Game” delete! If you love that term, please put into footnote.

5. SI p.11: why $p_i^* \in (0, 0.293)$?

6. SI p.12-13: what is difference E(KE) – P(KE), E(DU) – P(DU), resp.? Is “predicted” not the same as “expected”?

Answer 6:

1. In the context of the LUG means that when you participate in an interaction, you do not know, ex-ante, whether the interlocutor is bilingual or monolingual. Similarly, your interlocutor does not have information about your linguistic type. This information is therefore private. Hence, there is uncertainty for the bilingual involved in the interaction. Once the interaction starts, and if you are a bilingual who chooses the strategy Reveal, then the information that you are bilingual is revealed and cease to be private. The uncertainty has disappeared. However, if two bilinguals meet and both play Hide, the information keeps being private, the uncertainty has not been resolved, and they will speak in the majoritarian language A.

2. We use the concepts, *"utility" and "perceptible utility gain",* simply as it is standard in such theoretical concept or model. However, we have extended the notation to offer readers of other fields a better understanding. Our notation is commonly used in measurement theory, games and decision theory, and were introduced by the corresponding authors who worked in those fields (see for instance (11), (12)). The concept "perceptible utility gain" is a psychophysical expression that is used in Measurement Theory. It means that, given the bounded discrimination capacities of the human mind, utility differences below the perception threshold are not distinguishable. Binary relations like similarity and indifference are the outcome of bounded human rationality.

In game theory, "payoff" and "von Neumann and Morgenstern (sorry!) utility" or just "utility" are used equivalently. In the context of utility theory, "benefit", "profit", "net benefit", refer to levels of utility or payoff to the bilingual player. Whenever one of those terms is introduced in our work, you will find the mathematical expression of the term. Such as profit in line 7, page 13. Net profit is described by equation (5), and so on. What matters is the mathematical expression of the term. It may happen that the same mathematical expression is called sometimes benefit and sometimes profit. But we know that they mean the same mathematically. Frustration cost means a "utility loss".

3. "Preference intensity": this concept is introduced in Assumption **A.3**, page 7 of the main text, line 283: "Note that $m(\alpha_i) > n$ shows that the preference intensity for using B decreases as α_i increases". Where $m(\alpha_i)$ denotes the payoff (or utility) obtained from speaking in B, and n is the payoff from speaking in A, a positive constant. The inequality $m(\alpha_i) > n > 0$ means that the bilingual of locality i prefers using language B. The intensity

of this preference is measured by the size of the difference $m(\alpha_i) - n$. Since the function $m(\alpha_i)$ is assumed to be decreasing in α_i, the preference intensity decreases as α_i increases in $(0, alpha_i^*)$.

4. We certainly understand your point but actually it is not that we fancy the term. We use it because in the game-theory community it is well established (so it is not that 'we' love the term. You can find it in Wikipedia as a popular game theory model). But we have added 'so-called' and put it in quotation marks. Let us explain: we show in SI that the expected payoff matrix of the LUG is symmetric. In game theory there is a classification of symmetric 2x2 games. After the normalization of the 2x2 symmetric matrix, there are four types of symmetric games, depending on the payoffs of the main diagonal (outside this diagonal all elements are 0). If the normalized payoffs of the main diagonal are both negative, which is the case of our expected payoff matrix, then it is said that has the structure of the Hawk-Dove Game. In game theory it is common to mention it when you face a symmetric matrix like this. Finally, notice that we do not use it in the main text to avoid misunderstandings.

5. Because it is the solution of the quadratic equation resulting from the inequality that you find two lines below in the text.

6. This is a really good question, and sometimes admittedly confusing. While it is true that 'expectation' would be the safer term from a statistical point of view, unfortunately, the much less specific terminology 'prediction' is by far more popular and wide-spread over different communities. Please allow us to omit here any mathematical explanation of the general differences between prediction and expectation in mathematical statistics; it might be sufficient for our purpose that in these models and in our context, it is - fortunately - the same. We have included a sentence in the revised version that clarifies this issue saying that here we can and will use the terms prediction and expectation synonymously. So the easy answer to your question is that in this paper, yes, it is the same. However, we first estimate the equation for conditional expectations, and then use these estimates to predict the entire street or daily use functions.

To conclude, we hope you would understand our difficult position of using a formal model and being accessible to a broad audience, from linguists, to mathematicians, physicists, psychologists and behavioural scientists. We have tried to 'smooth' the text to be open to all those potential readers.

Thanks you for your work as a highly interested reviewer of our manu-

script.

References

1. Abrams and Strogatz (2003). Modeling the dynamics of language death. Nature, 424:900.

2. Kandler, A., Unger, R., and Steel, J. (2010). Language shift, bilingualism and the future of Britain's Celtic languages. Philos Trans R Soc Lond B Biol Sci, 365:3855-3864.

3. Patriarca, M., Castello, X., Uriarte, J.R., Eguiluz, V., and San Miguel, M. (2012). Modeling two language competition dynamics. Adv Complex Sys, 15:1-15.

4. Minett, J. and Wang, W.-Y. (2008). Modelling endangered languages: the effects of bilingualism and social structure. Lingua, 118:19-45.

5. Stauffer, D. and Schulze, C. (2005). Microscopic and macroscopic simulation of competition between languages. Phys Life Rev, 2:89-116.

6. Zhang, M. and Gong, T. (2013). Principles of parametric estimation in modeling language competition. Proc Natl Acad Sci USA, 110:9698-9703.

7. Fort, J. and Perez-Losada, J. (2012). Front speed of language replacement. Hum Biol, 84:755-772.

8. Isern, N. and Fort, J. (2014). Language extinction and linguistic fronts. J R Soc Interface, 11:1-9.

9. Sole, R., Corominas-Murtra, B., and Fortuny, J. (2010). Diversity, competition,extinction: the ecophysics of language change. J R Soc Interface, 7:1647-1664.

10. Prochazka, K. and Vogl, G. (2017). Quantifying the driving factors for language shift in a bilingual region. Proc Natl Acad Sci USA, 114:4365-4369.

11. Aizpurua, J.M., Nieto, J, and Uriarte, J.R. (1990). Choice procedure consistent with similarity relations. Theory and Decision 29: 235-254.

12. Aizpurua, J.M., Ichiishi, T., Nieto, J, and Uriarte, J.R. (1993). Similarity and preferences in the space of simple lotteries" Journal of Risk and Uncertainty, 6:289-297.
