# Supplementary material for: Correction: A behavioural model of minority language shift: Theory and empirical evidence
Source: PLoS One. 2021 Jul 9;16(7):e0254625. doi: 10.1371/journal.pone.0254625 (PMC8270155; doi:10.1371/journal.pone.0254625)
Supplement: S1 File — This file includes the authors’ response to reviewers for the first round of revisions. (PDF) [file pone.0254625.s001.pdf]

Additional Editor Comments:

Both reviewers agree that this paper studied an interesting and important question. However, they feel that the mathematical model is too complicated and difficult to understand. Since target readers are linguists, the author may provide some detailed explanations for background knowledge, e.g., evolutionary game method, and discuss why this method is better than other existing methods. Furthermore, since the model has several parameters, the author should provide intuitions for why these parameters are necessary and how they work.

ANSWER TO THE EDITOR

We appreciate very much your comments.

Note that some of the answers to your questions will be included too in the answers to the Reviewers #1 and #2, and in the revised version of the paper. Please read the answers to both Reviewers.

1. *Target readers.*

Answer 1:
It is true that linguists and sociolinguists are a group interested in language competition and shift (to name a few, see (1), (2)). But we must bear in mind that during the last decades, a research field of language competition and shift has been developed mainly by mathematicians and physicists, proposing mathematical models to study the issue. Many of those works are referenced in our paper. We intend to attract readers from different fields and research areas.
To this end, in the Introduction of the revised version, we avoid the use of technicalities and insist more on why evolutionary game theory is the natural framework to study interactive language decisions in the considered bilingual societies. Our article shows that repeated language choices made by bilinguals lead them to converge in to a linguistic convention as an evolutionary stable equilibrium. To understand this result, we introduce a simple example, **The Rules of the Road Game,** to help shed light on the mechanics of this dynamic result. The example allows us to introduce, step-by-step, the basic game-theoretic notions used in the work: Strict Nash Equilibrium and Evolutionary Stable Strategy as an emerging Convention. We hope that with this additional information, linguist and sociolinguist readers may smoothly go through the article and get, at least, its main message.
But our work has additional potential readers. Since we propose a behavioural model of language shift, our goal is to attract as well researchers from different fields of behavioural sciences, such as psychology, economics and cognitive sciences.

2. *Provide some detailed explanations for background knowledge, e.g., evolutionary game method*

Answer 2:

As it is said in the **Introduction** of the revised manuscript, the previous literature on mathematical models of language competition and shift did not consider bilingual individuals as decision makers. As a consequence, language shift was not seen as the outcome of their language choices. Hence, the introduction of behavioural assumptions and bilingual individuals as adaptive decision maker remains a challenge.

The following lines on background knowledge are summarized in the Introduction of the revised version and the advantages of formal approach is implicitly argued in the main text.

- *Game theory*:
  In the bilingual societies considered in the paper -where a majoritarian language A and a minoritarian one B are linguistically distant and in close contact- the nature of the choice of language is interactive. This is because the chosen language will serve the purpose of communication in interactions where bilinguals and monolinguals (who do not understand language B) might participate. As a result, the language B chosen by the bilingual might not be the actual language used in the interaction. Note that this is not so in a monolingual society (i.e., a society where it is spoken only one language). Game theory and, in particular, our Language Use Game model (LUG), provide the natural framework to introduce behavioural assumptions to study the interactive language choice behaviour of bilinguals.
- *Evolutionary game theory*:
  The replicator dynamics equation (RD) attached to the LUG is the appropriate framework to model how the repeated language choices made by bilinguals in every interaction. The repeated choices give rise to a language use dynamics leading to the building of linguistic conventions. The Loka-Volterra differential equations used in biology and in some models of language competition model have not the advantages of the RD. The former is merely a statistical account of animal competition (predator and prey) used as an allegory for language competition. Instead, as it is mentioned in the Introduction, the RD can be derived in social contexts from imitation and trial-and-error-learning-processes. Thus, linguistic conventions are the long-run outcomes of the language use dynamics derived from their continuous language choices. This convergence is shown by the theorem presented in our paper.

- *The advantage of using a formal model*:
  It allows us to establish functional relations between several relevant elements. More specifically, let us consider a location i (i in the set of all municipalities where language B is spoken) with a proportion α_i of bilinguals at a certain moment of time. The game theoretical approach allow us to relate the utility of speaking B, $m(\alpha_i)$, the utility of using language A, n, the frustration cost of being forced to use A, $c(\alpha_i)$, the net benefit of speaking the minority language B, $(m(\alpha_i) - n) - c(\alpha_i)$ and the aspiration function, $S(\alpha_i)$, with the proportion of bilinguals who would play the strategy *Reveal* in equilibrium, $p_i^* = p^*(\alpha_i)$. Notice that the aspiration function, $S(\alpha_i)$, will appear in the econometric specification of p_i^*. Equation (1) of our work shows that p_i^* is the global asymptotic attractor of the replicator equation and the unique evolutionary

stable Nash equilibrium (i.e. an ESS equilibrium) of the LUG. That is, p_i^* is the linguistic convention of municipality i, given α_i.

- $$p_i^* = p^*(\alpha_i) = 1 - \frac{(1-\alpha_i)c(\alpha_i)}{\alpha_i(\,m(\alpha_i)-n)} \qquad (1)$$

This equation predicts the rate of language shift in municipality i when its proportion of bilinguals is α_i. Then, taking into account all the municipalities of the country, we can build a function showing how $p_i^* = p^*(\alpha_i)$ changes with the changes in $\alpha_i \in (0,1)$. This result could not be possible to obtain using the non-formal analytical tools of sociolinguistics (e.g., (1) and (2)). This is the advantage of using the formal approach provided by evolutionary game theory.

3. *....and discuss why this method is better than other existing methods.*

Answer 3:
The answer to this question is covered in the revised main text both in the **Introduction** and in the last section of **Discussion and Conclusions.**
How does our work relate to other formal approaches to language competition and shift dynamics?

3.1 Imperfect Information on linguistic types (bilingual or monolingual):
None of the formal models proposed in (1)-(12) can handle the uncertainty derived from such imperfection. Models not viewing bilinguals as decision makers would not be able to deal with present day advanced sociolinguistic contexts where bilinguals are *uncertain* to recognize each other due to *imperfect information* of linguistic types. As it is shown in our work, game theory can perfectly deal with interactive language choices made under imperfect information.

3.2. Language prestige:
In the societies under consideration, bilinguals struggled to get B as official language, and a great proportion of them keep on in their quest for adapting the laws to allow equal opportunities in both languages. This means that *for the bilinguals, the languages A and B have the same status and equal prestige*. Hence, the shift of language B cannot be explained by parameters based on the *perceived value* of language B or its socioeconomic status. This parameter was introduced by the influential work of Abrams and Strogatz (3), and it is used by almost all the extensions of (3) (for example, (6); see also the literature arising from (3) in the survey (5)). Thus, the language prestige parameter is analytically useless in the bilingual societies considered in our work.

3.3. Reaction-Diffusion Models:
Bilingual education, economic development and social and geographical mobility in the societies under consideration, have shaped compact mixed populations of monolinguals and bilinguals across the country, particularly in urban areas, which are demographically and economically the most relevant ones. That is, there are no geographical concentrations of speakers of language A in one area and concentrations of speakers of B in another area.
However, the reaction-diffusion models could be analytically relevant in *linguistically segmented areas*, e.g. (4) (9) (10) (12). These models will usually deal with situations where B is spoken in a geographical area assumed to be rural or less developed and less

populated prone to be linguistically invaded by the language A spoken by a more powerful neighbour society. A linguistic wave front would be located in the weaker society gradually moving in the space, displacing the local use of their vernacular language. But this is not the situation in the societies under consideration. Hence, models based on travelling waves and linguistic fronts, are of no use here.
Our model can handle this situation as follows. We assume that the LUG is played in every municipality by its local population of bilinguals. We show that the empirical version of the equilibrium equation (1), $\alpha_i \in (0,1)$, is a strictly increasing and convex function that predicts the use of Basque in each municipality, in a given survey year. If we take all the surveys of, say, the Basque language (years 1993, 1997, 2001, 2006, 2011, 2016), we show that those predictions fit well the data of B use in every surveyed year. Then if we take all the survey years, the set of equilibrium functions, one function for each year, will predict and trace back the changes that have occurred in each municipality during those years. Hence, the LUG model can provide a space and temporal account of the changes of language B use at each municipality.

4. *Since the model has several parameters, the author should provide intuitions for why these parameters are necessary and how they work.*

Answer 4:
Our model has one variable, the observed proportion of bilinguals of each municipality, denoted $\alpha_i \in (0,\ 1)$, and three regression parameters, denoted β_2, β_3, and either $\widetilde{\beta_1}$ or $\overset{\frown}{\beta_1}$. The parameters are estimated from the data, **they are not pre-set by us**. The parameter estimates are presented in Tables 1, 2 and 3 of the Supplementary Information. The regression parameters are needed for the empirical specification of the functions entering equilibrium function $p_i^* = p^*(\alpha_i)$. That is, we need to specify the functions $m(\alpha_i)$, $c(\alpha_i)$ and $S(\alpha_i)$ having in mind their assumed properties (in assumption A.3). This is explained in section 6 of the Supplementary Information.
In the specification process, these parameters are unknown. When the regression is performed we then estimate their values, and for the model to make sense we work with $\widetilde{\beta_1} > 0$, $\overset{\frown}{\beta_1} > 0$ and $\beta_3 \in (0,1)$. Their estimations are presented in Tables 1, 2 and 3 of the Supplementary Information.

Remark: see also ANSWER 6 to Reviewer #1.

References:

1.Fishman, J. (1991). Reversing Language Shift. Theoretical and Empirical Foundations of Assistance to Threatened Languages. Multilingual Matters. Clevedon, UK.
2. Crystal, D. (2000). Language Death. Cambridge Univ Press. Cambridge,mUK.
3. Abrams and Strogatz (2003). Modeling the dynamics of language death. Nature, 424:900.
4. Kandler, A., Unger, R., and Steel, J. (2010). Language shift, bilingualism and the future of Britain's Celtic languages. Philos Trans R Soc Lond B Biol Sci, 365:3855-3864.
5. Patriarca, M., Castello, X., Uriarte, J.R., Eguiluz, V., and San Miguel, M. (2012). Modeling two language competition dynamics. Adv Complex Sys, 15:1-15.

6. Minett, J. and Wang, W.-Y. (2008). Modelling endangered languages: the effects of bilingualism and social structure. Lingua, 118:19-45.
7. Stauffer, D. and Schulze, C. (2005). Microscopic and macroscopic simulation of competition between languages. Phys Life Rev, 2:89-116.
8. Zhang, M. and Gong, T. (2013). Principles of parametric estimation in modeling language competition. Proc Natl Acad Sci USA, 110:9698-9703.
9. Fort, J. and Perez-Losada, J. (2012). Front speed of language replacement. Hum Biol, 84:755-772.
10. Isern, N. and Fort, J. (2014). Language extinction and linguistic fronts. J R Soc Interface, 11:1-9.
11. Sole, R., Corominas-Murtra, B., and Fortuny, J. (2010). Diversity, competition,extinction: the ecophysics of language change. J R Soc Interface, 7:1647-1664.
12. Prochazka, K. and Vogl, G. (2017). Quantifying the driving factors for language shift in a bilingual region. Proc Natl Acad Sci USA, 114:4365-4369.

Reviewer #1: This paper proposes language use game and uses Nash equilibrium theory and evolutionary stable solution to estimate the outcome of language shift in a community with a monolingual A and bilingual A & B. The authors briefly described the method and math behind and conducted experiments using theoretical setting and a setting supported by real data.

My general feeling is that the adoption of equilibrium based math in language shift modeling is a great innovation, though it requires deep math knowledge to understand, which may drive away linguists who are interested in the topic of language shift. To better understand the relevant concepts, it would be great that the authors provide some simple examples of ESS and background knowledge, before jumping directly to the complex math model. Keep in mind that the readers of your paper may not come from physics or math, but historical/contact linguistics, who may not have sufficient knowledge to understand your current writing, no understanding, no use.

In addition, why using such math, whether it is because it has some advantages that other approaches cannot provide, or this method can avoid some parameters that are hard to estimate from real data. The authors mentioned something similar only in the conclusion section (why other models cannot address the issues you raised), it would be great to move that part early in the introduction, and also, additional justification is needed.

Finally, given the parameters in the model, is it possible to conduct an analysis on the effect of major parameters, like alpha or alpha star, i found the current values are set pretty arbitrary.

minor issues:
line 23, delete ","
line 40, 'european'--> 'European'
the 2nd paragraph in introduction is too long, and incorporate many points, better to separate each point into one paragraph.
line 126: why street use of Basque is short for KE?
line 151: "could is it" -> 'is it'

line 470: citefortEtal2012 must be changed to reference

ANSWER to Reviewer #1:

We appreciate very much your comments.

"*Keep in mind that the readers of your paper may not come from physics or math, but historical/contact linguistics, who may not have sufficient knowledge to understand your current writing, no understanding, no use*".

Answer 1:
It is true that linguists and sociolinguists are a group interested in language competition and shift (to name a few, see (1), (2)). But we must bear in mind that during the last decades, a research field of language competition and shift has been

developed mainly by mathematicians and physicists, proposing mathematical models to study the issue. Many of those works are referenced in our paper.
We intend to attract readers from both sides. In the Introduction of the revised version, we avoid the use of technicalities and insist more on why evolutionary game theory is the natural framework to study interactive language decisions in the considered bilingual societies. Our article shows that repeated language choices made by bilinguals lead them to converge in to a linguistic convention as an evolutionary stable equilibrium. We introduce a simple example, reproduced below, **The Rules of the Road Game,** to help shed light on the mechanics of this dynamic result. The example allows to introduce, step-by-step, the basic game-theoretic notions used in the work: Strict Nash Equilibrium and Evolutionary Stable Strategy as an emerging Convention. Then, we hope that the linguist and sociolinguist readers may smoothly go through the article and get its main message.
Since the work proposes a behavioural model of language shift, our goal is to attract as well researchers from different fields of behavioural sciences, such as psychology, economics and cognitive science.

"To better understand the relevant concepts, it would be great that the authors provide some simple examples of ESS and background knowledge, before jumping directly to the complex math model."

Answer 2:
We have made some changes in the **Introduction** explaining step-by-step why game theory and evolutionary game theory are the natural analytical frameworks to study interactive language choices in the bilingual societies under consideration in our work. The following lines are a contained in the Introduction.

- *Game theory*:
  The societies under study have the following three relevant features: the languages A and B are linguistically distant. There are no geographical or any other type of barriers between bilinguals and monolinguals. They are in close contact. Finally, people's linguistic type is private information. Under these conditions, the language decisions taken by bilinguals have an interactive nature. That is, since the chosen language will serve the purpose of communication in interactions where bilinguals and monolinguals might meet, the choice of B made by a bilingual might not be the actual language that will be used in the interaction. Note, as well, that this type of interactive decisions hardly occurs in a monolingual society (i.e., a society where it is spoken only one language). Game theory and, in particular, our game named the Language Use Game model (LUG, henceforth), is the natural framework to study interactive language decisions, allowing us to introduce behavioural assumptions.
- *Evolutionary game theory*:
  The replicator dynamics equation (RD) attached to the LUG is the appropriate framework to model how the repeated language choices made by bilinguals in every interaction. The repeated choices give rise to a language-use dynamics leading to the building of linguistic conventions. The Loka-Volterra differential equations used in biology and in some models of language competition model

have not the advantages of the RD. The former is merely a statistical account of animal competition (predator and prey) used as an allegory for language competition. Instead, as it is mentioned in the Introduction, the RD can be derived in social contexts from imitation and trial-and-error-learning-processes. Thus, linguistic conventions are the long-run outcomes of the dynamics derived from their continuous language choices. This convergence is shown by the theorem presented in our paper.

Answer 3:

The following example is intended to provide an intuitive idea of the notions of Nash equilibrium and Evolutionary Stable Strategy equilibrium as an emerging Convention. The example will be placed in the main text as a subsection of METHODS, just before the subsection The Language Use Game Model.

EXAMPLE: **The Rules of the Road Game**

Let us consider the game of which side of the road to drive on, whether left side or right side (for more details, see (13)). The game is played by two oncoming vehicles. Since drivers want to avoid accidents, they will coordinate on the same side of the road. Hence, this is a coordination game. Drivers have no incentives to deviate from coordinating on the same side because they are strictly better off than being involved in an accident. This is the basis for the notion of strict Nash equilibrium. Hence, this coordination game has two strict Nash equilibria (Left, Left) and (Right, Right). These equilibria are two conventions. But which of the two equilibria will be chosen? which is the same as asking which of the two conventions, driving on the right or driving on the left, will be adopted by the society? To answer this question, we should view how the game evolves historically by the repetition of the interaction. We can think of the game being played by a large population. Each time a different pair of conductors will play the game and may remember how the interaction was solved. As the game is repeated over and over by many people, one particular way of solving the interactions, say (R, R), takes prominence in the population, driving out the other one, (L, L). This process of emerging the convention is by accumulation of precedent. In our view, this mechanism in which imitation plays an important role might explain the linguistic convention built by bilinguals in the present paper.

But a convention may become established through a different mechanism. Initially, when traffic was scarce, some area of the country might adopt, by local custom, say, the left convention, while other areas of the country adopt the right convention. As traffic grew in volume the evolution of those conventions will be affected by sudden changes in the law regulating the traffic at the national level. These changes will also consider the chosen conventions made by the neighboring countries. Finally, the society decides politically to stablish one convention. These two mechanisms are not mutually exclusive. Note that in terms of welfare both conventions, (R, R) and (L, L), are equally good. What the established convention does is to solve the indeterminacy. That is, we may define a convention that has emerged historically as *an equilibrium that everybody expects* in interactions that have more than one equilibrium, as in the present game. In other words, expectations and behaviors are in equilibrium in

the established convention. Therefore, the convention that has emerged and has been adopted by the society, is an evolutionary stable strategy of the Rules of the Road Game.
In the Language Use Game we show that bilinguals converge into an evolutionary stable equilibrium in which they do not all play the strategy that will coordinate them in speaking language B.

"In addition, why using such math, whether it is because it has some advantages that other approaches cannot provide, or this method can avoid some parameters that are hard to estimate from real data. The authors mentioned something similar only in the conclusion section (why other models cannot address the issues you raised), it would be great to move that part early in the introduction, and also, additional justification is needed."

Answer 4:

The advantage of using a formal model

The mathematics of evolutionary game theory enables us to introduce a behavioural approach to understand minority language shift. We establish formally what sociolinguists say, that minority language shift is the outcome of the language choices made by bilingual individuals, in a context of language competition.
We should also say that using the non-formal analytical tools of sociolinguistics (e.g., (1) and (2)) we would not be able to relate the utility of speaking B, $m(\alpha_i)$, the utility of using language A, n, the frustration cost of being forced to use A, $c(\alpha_i)$, the net benefit of speaking the minority language B, $(m(\alpha_i) - n) - c(\alpha_i)$ and the aspiration function, $S(\alpha_i)$, with the proportion of bilinguals who would play the strategy *Reveal* in equilibrium, $p_i^* = p^*(\alpha_i)$. Notice that the aspiration function, $S(\alpha_i)$, will appear in the econometric specification of p_i^*. Equation (1) of our work shows that p_i^* is the global asymptotic attractor of the replicator equation and the unique evolutionary stable Nash equilibrium (i.e. an ESS equilibrium) of the LUG. That is, p_i^* is the linguistic convention of municipality i, given α_i.

- $$p_i^* = p^*(\alpha_i) = 1 - \frac{(1-\alpha_i)c(\alpha_i)}{\alpha_i(m(\alpha_i)-n)} \qquad (1)$$

With a no mathematical model we would not be able to get equation (1) that predicts the rate of language shift in municipality i when its proportion of bilinguals is α_i. Taking into account all the municipalities of the country, we then can build a function showing how $p_i^* = p^*(\alpha_i)$ changes with the changes in $\alpha_i \in (0,1)$.

Answer 5:

The advantages with respect other models:

To avoid a long Introduction to main text, what we say in the following lines will be placed in the Conclusion section.

1. Our model can handle imperfect information and uncertainty about linguistic types (bilingual or monolingual):
None of the formal models proposed in (1)-(12) can handle the presence of such imperfection. Models not viewing bilinguals as decision makers would not be able to deal with present day advanced sociolinguistic contexts where bilinguals are *uncertain* to recognize each other due to *imperfect information* of linguistic types. As it is shown in our work, evolutionary game theory can perfectly deal with interactive language choices made under imperfect information.

2. Language prestige:
In the societies under consideration, bilinguals struggled to get B as official language, and a great proportion of them keep on in their quest for adapting the laws to allow equal opportunities in both languages. This means that *for the bilinguals, the languages A and B have the same status and equal prestige*. Hence, the shift of language B cannot be explained by parameters based on the *perceived value* of language B or its socioeconomic status. This parameter was introduced by Abrams and Strogatz (3), and is used by almost all extensions of (3) (for example, (6); see also the literature arising from (3) in the survey (5)). Thus, the language prestige parameter is *analytically useless* in the bilingual societies considered in our work.

3. Reaction-Diffusion Models:
Bilingual education, economic development and social and geographical mobility in the societies under consideration, have shaped tight mixed populations of monolinguals and bilinguals across the country, particularly in urban areas, which are demographically and economically the most relevant ones. That is, there are no geographical concentrations of speakers of language A in one area and concentrations of speakers of B in another area. In some models the latter would be assumed to be rural or less developed, prone to be linguistically invaded, and where the linguistic wave front would be located. However, for the reaction-diffusion models to be analytically relevant, it is needed linguistically segmented areas, e.g. (4) (9) (10) (12). Hence, models based on travelling waves and linguistic fronts, are of no use here. Our model can handle this situation as follows. We assume that the LUG is played in every municipality by its local population of bilinguals. We show that the empirical version of the equilibrium equation (1), $\alpha_i \in (0,1)$, is a strictly increasing and convex function that predicts the use of Basque in each municipality, in a given survey year. If we take all the surveys of, say, the Basque language (years 1993, 1997, 2001, 2006, 2011, 2016), we show that those predictions fit well the data of B use in every surveyed year. Then if we take all the survey years, the set of equilibrium functions, one function for each year, will predict and trace back the changes that have occurred in each municipality during those years. Hence, the LUG model can provide a space and temporal account of the changes of language B use at each municipality.

"Finally, given the parameters in the model, is it possible to conduct an analysis on the effect of major parameters, like alpha or alpha star, i found the current values are set pretty arbitrary."

Answer 6:
This is a misunderstanding. In fact, all parameter in the model are estimated from the data, **they are not pre-set by us**. The parameter estimates are presented in Tables 1, 2 and 3 of the Supplementary Information. In contrast, the $\alpha_i \in (0,\ 1)$ are not parameters, but our observed data. It represents the percentage of bilinguals in a given community i in year t. That is, our model has one (observational) variable $\alpha_i \in (0,\ 1)$ and three regression parameters, denoted β_2, β_3, and either $\widetilde{\beta_1}$ or $\overset{\frown}{\beta_1}$. These parameters are used for the empirical specification of the functions $m(\alpha_i)$, $c(\alpha_i)$ and $S(\alpha_i)$, having in mind that they must satisfy their assumed properties (see A.3). The building of empirical version of the model is explained in section 6 of the Supplementary Information. This is an important part of our analysis since the empirical version of the model is needed to counter-check the predictions of the model with observational data
These regression parameters are unknown, but for the model to make sense we work with $\widetilde{\beta_1} > 0$, $\overset{\frown}{\beta_1} > 0$ and $\beta_3 \in (0{,}1)$.
As we do not consider panel models, but only cross sectional regression, we can skip index t for time but estimate the parameters beta for each year and minority language separately, pooling only the communities inside each linguistic area (like Wales) for a given year.
As it is common in the literature, we further simplify notation by skipping index i (for community) when discussing the statistical model in general.
Finally, α^* or α_i^* refer to fictitious, non-observable numbers inside our theoretical model. They denote the aspiration of the country and the aspiration of municipality i, respectively, indicating the percentage of bilinguals at which the country and the municipality would no longer consider language B as a threatened minority language. The idea of such fictitious number (say, threshold) helps us to justify the parametrization of the pay-off function.
We hope this explanation has clarified your doubts.

"why street use of Basque is short for KE?"

ANSWER 7:
The translation into Basque of "Street Use" is "Kale Erabilera". In this case, we wanted to use KE instead of SU, just to give a little presence to the minority language. We will explain it in the text.
The rest of the typos and suggestions are done.

References:

1.Fishman, J. (1991). Reversing Language Shift. Theoretical and Empirical Foundations of Assistance to Threatened Languages. Multilingual Matters. Clevedon, UK.

2. Crystal, D. (2000). Language Death. Cambridge Univ Press. Cambridge,mUK.
3. Abrams and Strogatz (2003). Modeling the dynamics of language death. Nature, 424:900.
4. Kandler, A., Unger, R., and Steel, J. (2010). Language shift, bilingualism and the future of Britain's Celtic languages. Philos Trans R Soc Lond B Biol Sci, 365:3855-3864.
5. Patriarca, M., Castello, X., Uriarte, J.R., Eguiluz, V., and San Miguel, M. (2012). Modeling two language competition dynamics. Adv Complex Sys, 15:1-15.
6. Minett, J. and Wang, W.-Y. (2008). Modelling endangered languages: the effects of bilingualism and social structure. Lingua, 118:19-45.
7. Stauffer, D. and Schulze, C. (2005). Microscopic and macroscopic simulation of competition between languages. Phys Life Rev, 2:89-116.
8. Zhang, M. and Gong, T. (2013). Principles of parametric estimation in modeling language competition. Proc Natl Acad Sci USA, 110:9698-9703.
9. Fort, J. and Perez-Losada, J. (2012). Front speed of language replacement. Hum Biol, 84:755-772.
10. Isern, N. and Fort, J. (2014). Language extinction and linguistic fronts. J R Soc Interface, 11:1-9.
11. Sole, R., Corominas-Murtra, B., and Fortuny, J. (2010). Diversity, competition,extinction: the ecophysics of language change. J R Soc Interface, 7:1647-1664.
12. Prochazka, K. and Vogl, G. (2017). Quantifying the driving factors for language shift in a bilingual region. Proc Natl Acad Sci USA, 114:4365-4369.
13. Young, H.P. (1996), The economics of convention, Journal of Economic Perspectives, Volume 10, No. 2:105–12

Reviewer #2: In my opinion this is an important attempt to understand linguistic behaviour of people in bilingual societies and may add to policy makers' decisions (and to the awareness of speakers of language B) when trying to save endangered languages.

My knowledge of game theory and statistics is not sufficient to judge the corresponding equations, but I feel they are O.K.

Some details:

Why not show data points of recorded observations in the figures in the main text?

Fig.2: I think the axis should read "fraction", not "%", same in figures in supp.info. Alpha, not alpa.

Line 470: Fort and Perez-Losada

Supp.info:

p.2, Ireland: delete "As for 1996 this distinction is not at all clear we skipped this wave in the revised version of our paper."

p.9: "..where ??? and Nij(t)=Ni represents the share of bilinguals of locality i playing pure strategy j at t (j = R; H)."

p.12: (***)

ANSWERS:

We appreciate very much your comments. These are the answers to each of your comments:

My knowledge of game theory and statistics is not sufficient to judge the corresponding equations

ANSWER 1:

The provide the basic knowledge for a smoother reading of the paper, we have made some changes in the Intro, explaining step-by-step why game theory and evolutionary game theory are the right framework to study the bilinguals' language choice behaviour. Further, before describing the Language Use Game, we have introduced an example where the concepts of strict Nash equilibrium and Evolutionary Stable Equilibrium as an emerging Convention.

EXAMPLE: **The Rules of the Road Game**

Let us consider the game of which side of the road to drive on, whether left side or right side (for more details, see Young (1996)). The game is played by two oncoming vehicles. Since drivers want to avoid accidents, they will coordinate on the same side of the road. Hence, this is a coordination game. Drivers have no incentives to deviate from coordinating on the same side because they are strictly better off than being involved in an accident. This is the basis for the notion of strict Nash equilibrium. Hence, this coordination game has two strict Nash equilibria (Left, Left) and (Right, Right). These equilibria are two rules or conventions. But which of the two equilibria will be chosen? which is the same as asking which of the two conventions, driving on the right or driving on the left, will be adopted by the society? To answer this question, we should view how the game evolves historically by the repetition of the interaction. We can think of the game being played by a large population. Each time a different pair of drivers will play the game and may remember how the interaction was solved. As the game is repeated over and over by many people, one particular way of solving the interactions, say (R, R), takes prominence in the population, driving out the other one, (L, L). This process of emerging the convention is by accumulation of precedent. In our view, this mechanism in which imitation plays an important role might explain the linguistic convention built by bilinguals in the present paper.

A convention may become established through a different mechanism. Initially, when traffic was scarce, some area of the country might adopt, by local custom, say, the left convention, while other areas of the country adopt the right convention. As traffic grew in volume the evolution of those conventions will be affected by sudden changes in the law regulating the traffic at the national level. These changes will also consider the chosen conventions made by the neighboring countries. Finally, the society decides politically to stablish one convention. These two mechanisms are not mutually exclusive. Note that in terms of welfare both conventions, (R, R) and (L, L), are equally good. What the established convention does is to solve the indeterminacy. That is, we may define a convention that has emerged historically as *an equilibrium that everybody expects* in interactions that have more than one equilibrium, as in the present game. In other words, expectations and behaviors are in equilibrium in the established convention. Hence, the convention that has emerged and has been adopted by the society, relative to the Rules of the Road Game, is an evolutionary stable strategy.

In the Language Use Game we show that bilinguals converge into an evolutionary stable equilibrium in which they do not all play the strategy that will coordinate them in speaking language B.

Some details:
Why not show data points of recorded observations in the figures in the main text?

ANSWER 2:

One could do that but then one has to reorganize the visual presentation of results. There are several reasons for our choice, the most important ones are the following:

To be concise, but as informative as possible, we thought it is more interesting to show for each linguistic area the development over years.

This makes it impossible to jointly show the data points inside Figure 2 (you cannot

show the point clouds of all the years in one picture, because then you see nothing!). Instead, we show the boxplots of the observations. For the common reader, together with the estimates relating alpha to language use, they are actually more informative than 11 pictures of point clouds (2 for Welsh, 3 for Irish, and 6 for Basque).

"Fig.2: I think the axis should read "fraction", not "%", same in figures in supp.info."

ANSWER 3:

We depict in the horizontal axis the observational variable alpha, which, all along the text, is referred to as the proportion of bilinguals. When in the text it is written down as $\alpha_i \in (0,1)$, it denotes the proportion of bilinguals of municipality i. We do not think there is a problem here since the proportion of bilinguals, denoted %, and the fraction of bilinguals in the total population of a municipality are mathematically equivalent.

Finally: all typos are corrected.

Reference:

Young, H.P. (1996), The economics of convention. Journal of Economic Perspectives, Volume 10, No. 2:105–12
